# Circular Dichroism Reveals Positive Cooperativity through Compensatory Motion in Stepwise Cage Metalation

**DOI:** 10.1021/acs.inorgchem.6c00988

**Published:** 2026-03-31

**Authors:** Melvin Raulin, Chiara Slaviero, Federico Begato, Giulia Licini, Cristiano Zonta

**Affiliations:** † Department of Chemical Sciences, University of Padova, Via F. Marzolo 1, 35131 Padova, Italy; ‡ CIRCC—Consorzio Interuniversitario per le Reattività Chimiche e la Catalisi, Via C. Ulpiani 27, 70126 Bari, Italy; § Aix Marseille Univ, CNRS, Centrale Med, ISM2, 13013 Marseille, France

## Abstract

Biological allosteric systems achieve functional control through long-range communication between distant binding sites. Here, we report that a symmetric chiral cage bearing two equivalent tris­(2-pyridylmethyl)­amine (**TPMA**) binding sites exhibits structural communication reminiscent of allosteric behavior during stepwise Zn­(II) coordination. NMR titrations reveal sequential metalation kinetics in which the second coordination step becomes comparatively slower in mixed solvents, consistent with electrostatic effects and structural preorganization. Circular dichroism (CD) spectroscopy tracks the progressive structural evolution of the cage, with five isodichroic points confirming clean interconversion between discrete species. TD-DFT calculations reproduce the experimental chiroptical evolution, and correlate the observed spectral changes with systematic geometric reorganization of the framework over distances exceeding 10 Å. This work illustrates the potential of CD spectroscopy as a diagnostic tool for probing allosteric-like mechanisms in synthetic supramolecular systems, with implications for heterometallic catalysis, responsive materials, and allosteric synthetic receptors.

## Graphical abstract

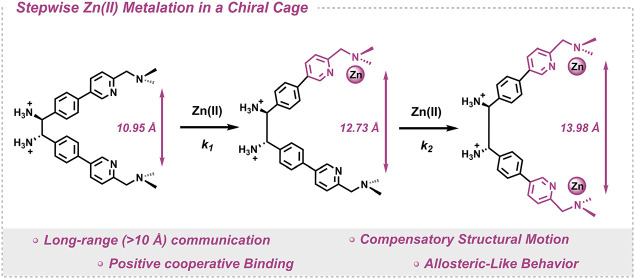

## Introduction

Nature’s metalloenzymes achieve fine functional control through precise positioning of multiple metal centers. However, replicating this level of control in symmetric synthetic systems remains a significant challenge in coordination chemistry.^1^ By combining different metal centers within a single molecular framework, such systems offer access to cooperative effects, complementary reactivity, and emergent properties that are not attainable in homometallic analogues.^2–4^ A wide variety of heterometallic complexes and assemblies have been successfully developed using asymmetric ligands, stepwise synthetic strategies, or preprogrammed coordination environments.^5–9^ In contrast, achieving similar control within symmetric homoditopic supramolecular scaffolds remains more challenging.^10–13^ In these systems, chemically equivalent binding sites often promote scrambling and rapid exchange, leading to statistical distributions or preferential formation of fully metalated species, and requiring careful control of assembly conditions to access defined heterometallic architectures.^14–16^

Tris­(2-pyridylmethyl)­amine (**TPMA**) ligands provide a particularly suitable platform to investigate metalation processes.^17^ Their polypyridyl nature enables the formation of well-defined complexes with a broad range of metal ions, while their intrinsic stereodynamic character leads to a propeller-like helical arrangement upon metal binding that is sensitive to subtle variations in the coordination sphere. As a consequence, different geometries are typically associated with distinct structural signatures, allowing metalation events to be readily monitored and differentiated within complex architectures.^18^ Over the past years, our group has developed a family of **TPMA**-based molecular cages assembled through dynamic covalent chemistry, which have served as versatile platforms for molecular recognition, stereodynamic amplification, and chiroptical sensing in confined environments.^19–27^ When incorporated into supramolecular cages, **TPMA** units provide access to symmetric architectures featuring two chemically equivalent binding sites within a confined environment. Such homotopic systems are particularly informative for probing metalation processes, as they allow the first and second coordination events to be distinguished within a common structural framework. At the same time, the equivalence of the binding sites renders these systems especially sensitive to kinetic and thermodynamic effects, as metal scrambling and rapid exchange can readily dominate the coordination behavior.

More recently, the synthetic approach was extended to a hydrolytically stable chiral cage combining imine condensation with a [3,3]-diaza-Cope rearrangement, enabling the incorporation of well-defined metal centers within a robust covalent framework.^28^ These systems were subsequently applied to catalysis, notably to light-driven hydrogen evolution mediated by confined cobalt-**TPMA** complexes.^29^ During these catalytic studies, the homotopic covalent chiral cage was found to provide clean access to a monometallic cobalt­(II) complex, as well as to heterometallic species upon coordination of a second metal center such as Zn­(II) or Cu­(II), a feature that was exploited to modulate catalytic performance.^30^

In this work, we investigate the origin of this unusual metalation behavior by combining NMR kinetics, CD spectroscopy, and DFT calculations. We show that the combined spectroscopic and computational approach serves as a structural reporter capable of tracking stepwise metalation in symmetric cages, revealing structural communication between the two metal-binding sites reminiscent of allosteric behavior, whereby coordination at one site influences the organization of a remote, chemically equivalent site separated by more than 10 Å. This approach provides insight into how coordination events propagate through confined supramolecular frameworks, with implications for the rational design of heterometallic assemblies and functionally responsive architectures.

## Results and Discussion

### NMR Analysis of Stepwise Zn­(II) Metalation

The **TPMA**-based cage ***S*** **-1**, previously reported as a chiral and hydrolytically stable architecture, was selected as a model system to investigate the kinetics of stepwise Zn­(II) incorporation. In our earlier work, titrations of either ***S*** **-1·Cl** with ZnCl_2_ or ***S*** **-1·OTf** with Zn­(OTf)_2_ revealed an identical two-step coordination sequence in which a transient monometalated species, **ZnH**_**4**_, formed en route to the fully metalated **ZnZn** complex.^30^ In both cases, **ZnH**_**4**_ was detected in situ together with ***S*** **-1** and **ZnZn**, and could not be isolated, whereas **ZnZn** appeared as the dominant species once two equivalents of Zn­(II) had been reached. Notably, the OTf^–^ system displayed enhanced resolution in the pyridyl region, allowing for the loss of equivalence between the two **TPMA** units in **ZnH**_**4**_ to be observed directly through a characteristic splitting of the α-pyridinic protons (ca. 8.8 ppm). This desymmetrization therefore provides a convenient spectroscopic handle for tracking the first metalation step in the OTf^–^ system (Figure 1).

**Figure 1:**
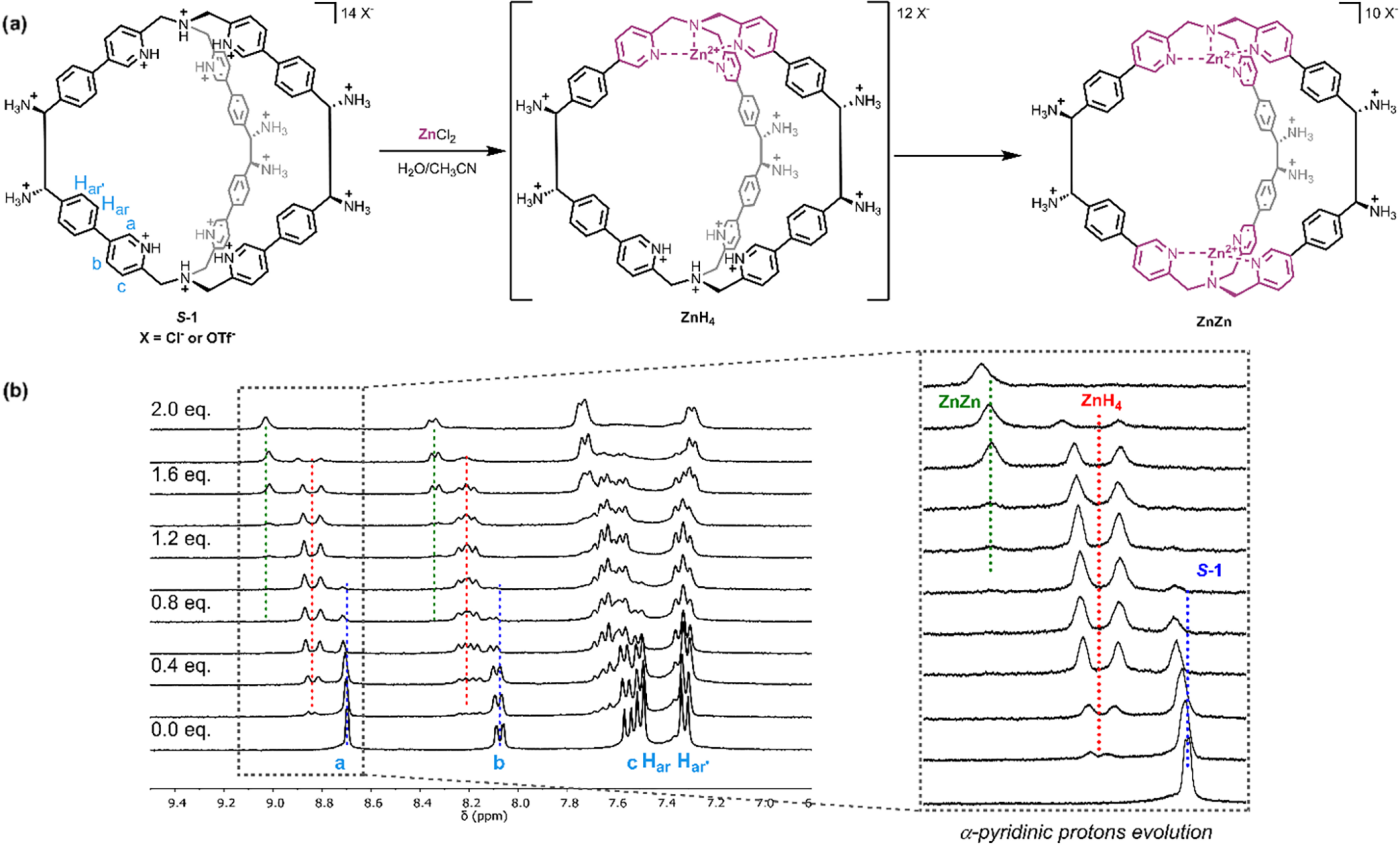
(a) Stepwise coordination of Zn­(II) by ***S*** **-1**, yielding the transient **ZnH**_**4**_ and the final **ZnZn**. (b) Partial ^1^H NMR spectra (D_2_O/CD_3_CN (1:1), 300 MHz, 298 K) illustrating the progressive coordination of ***S*** **-1** (with OTf^–^ as counterions) upon incremental addition of Zn­(OTf)_2_ (0 to 2 equiv).

On this basis, the kinetics of metalation were investigated for the ***S*** **-1·OTf**/Zn­(OTf)_2_ system, at increasing Zn­(II) concentrations and in different solvent mixtures (see Section S2, Figures S1–S22). In D_2_O, both coordination events occur rapidly and **ZnH**_**4**_ is observed as a minor transient species, with signals appearing at early reaction times and being quickly consumed to form the bimetallic species. A contrasting behavior is observed in CD_3_CN/D_2_O (1:1), where the second metalation step becomes significantly slower, leading to an accumulation of the monometallic intermediate **ZnH**_**4**_.

Kinetic fitting of the concentration profiles yielded apparent rate constants of *k*_1_ = 0.160 (±0.021) mM^–1^ min^–1^ and *k*_2_ = 0.100 (±0.023) mM^–1^ min^–1^ in CD_3_CN/D_2_O (1:1), compared to *k*_1_ = 0.774 (±0.090) and *k*_2_ = 0.573 (±0.037) mM^–1^ min^–1^ in D_2_O (see Figure S23). It is important to note that the first metalation event involves two identical coordination sites, requiring a statistical correction factor of 2. Upon applying this correction, the intrinsic rate constants become *k*_1_/2 = 0.387 (±0.045) mM^–1^ min^–1^ and *k*_1_/2 = 0.080 (±0.011) mM^–1^ min^–1^ for D_2_O and CD_3_CN/D_2_O (1:1), respectively. This reveals that the second metalation step is actually faster than the first in both solvent systems, with *k*_2_/*k*_1_ ratios of approximately 1.5 in D_2_O and 1.3 in CD_3_CN/D_2_O. This apparent positive cooperativity suggests that the initial Zn­(II) coordination facilitates the binding of the second metal ion, consistent with a kinetically cooperative stepwise process inferred from the NMR data, possibly through conformational preorganization of the ligand scaffold. The cooperative effect is more pronounced in pure aqueous solution.

While ^1^H NMR reveals the stepwise metalation kinetics, it provides no structural insight into the observed behavior. We therefore turned to UV–vis and CD spectroscopy, which exploit the inherent chirality of the cage, to probe whether Zn­(II) incorporation triggers measurable structural reorganization and, combined with DFT, to correlate chiroptical response with cage architecture.

### Chiroptical and UV–Vis Evolution

#### Circular Dichroism Titration

CD spectroscopy is a powerful and reliable tool for investigating supramolecular organization in chiral systems, as chiroptical techniques are generally highly sensitive to subtle conformational and intermolecular effects.^31^ Its sensitivity to chromophore orientation, helicity, and excitonic coupling makes it ideally suited to detect subtle structural reorganizations that may not be evident through other spectroscopic methods.^32^ As a result, even subtle geometric variations can give rise to marked changes in band shape, sign, or intensity.^33–36^ In metal-binding chiral assemblies, coordination of metal ions can act as a trigger for such organizational modifications, even when the associated structural changes are not readily captured by NMR spectroscopy alone, as also observed in other chiral host systems upon guest binding.^37^

Polypyridyl ligand such as **TPMA** offer well-established examples of this behavior.^38,39^ Although achiral in their free state, **TPMA** units adopt propeller-like helical conformations upon metal coordination, giving rise to characteristic chiroptical signatures. When embedded within a preorganized chiral environment such as cage ***S*** **-1**, these stereodynamic motifs act as sensitive reporters of coordination-induced structural reorganization, allowing coordination events to be tracked through changes in the CD spectrum.^18,40,41^ For this reason, we investigated the UV–vis and CD evolution of ***S*** **-1** during metalation to assess how the sequential Zn­(II) incorporation identified by NMR is reflected at the structural level.

Upon incremental addition of ZnCl_2_ (0–2 equiv) to a solution of ***S*** **-1** in a mixture of MeCN/H_2_O (1:1), the CD spectra display a continuous evolution characterized by five isodichroic points at λ = 208, 222, 239, 262, and 284 nm (Figure 2a, left). This behavior is consistent with an interconversion between discrete species, in agreement with the two-step metalation sequence observed by ^1^H NMR.^22,42^

**Figure 2:**
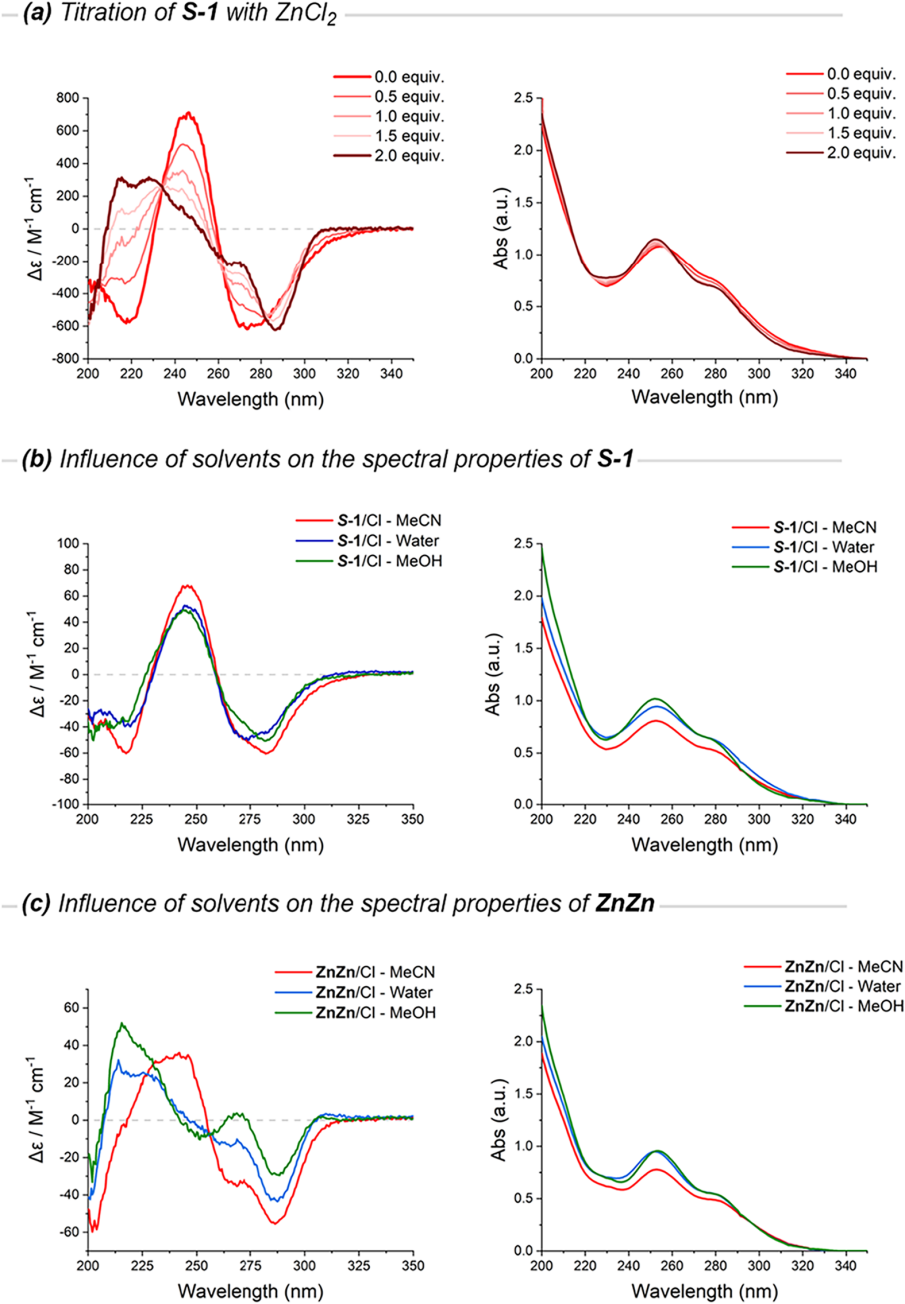
Electronic circular dichroism (left) and UV–vis (right) spectra. (a) Stepwise titration of ***S*** **-1** with ZnCl_2_ in MeCN/H_2_O (1:1). (b) Influence of solvents (MeCN, MeOH, H_2_O) on the spectral properties of ***S*** **-1**. (c) Influence of solvents (MeCN, MeOH, H_2_O) on the spectral properties of **ZnZn**.

The free cage ***S*** **-1** exhibits a well-defined bisignate CD pattern (negative–positive-negative), with extrema at 220, 243, and 277 nm. Upon Zn­(II) addition, the overall amplitude decreases and the spectral profile becomes increasingly complex, confirming the complexation is taking place. At intermediate Zn­(II) loadings (0.5–1.5 equiv), a broadened positive region (220–245 nm) and a less intense long-wavelength minimum (∼282 nm) emerge, consistent with the accumulation monozinc intermediate **ZnH**_**4**_ previously detected by NMR. Upon reaching 2 equiv of Zn­(II), the CD spectrum displays a distinct pattern which differs markedly from that of ***S*** **-1** and **ZnH**_**4**,_ featuring two positive bands at 222 and 230 nm and one negative band near 282 nm.

These chiroptical features originate from electronic transitions within the π-conjugated framework of the TPMA chromophores, primarily involving π–π excitations of the pyridyl and phenyl units. In such tripodal polypyridyl systems, metal coordination induces a well-defined helical arrangement of the ligand arms, which gives rise to characteristic CD signatures.^39^ The observed bisignate CD patterns are characteristic of exciton coupling between these chromophores, arising from their well-defined chiral spatial arrangement within the cage. As a result, the sign, intensity, and splitting of the CD bands are highly sensitive to the relative orientation and electronic interaction of these units. The progressive changes observed upon Zn­(II) coordination therefore reflect modulation of interchromophoric coupling induced by structural reorganization of the cage framework.

The corresponding UV–vis spectra evolve similarly (Figure 2a, right), with a modest blue-shift of the main absorption (∼255 → 252 nm) and an isosbestic point at 345 nm, consistent with clear interconversion between discrete species. This close correspondence between UV–vis and CD evolution indicates that both electronic and chiroptical responses reflect the same structural progression.

### Solvent and Counterion Effects on (Chir)­Optical Signatures

To assess whether the observed chiroptical signatures reflect only intrinsic cage architecture or are also influenced by solvent and counterion effects, we systematically measured UV–vis and CD spectra of both ***S*** **-1** and **ZnZn** across varying solvent polarities (H_2_O, MeOH, MeCN) and counterions (Cl^–^, OTf^–^) (Figure 2b,c).

In the UV–vis spectra the main π–π* absorption (characteristic of **TPMA** chromophores^43^) remains centered at 245–255 nm across all solvents, with only modest intensity modulations. Counterion substitution (Cl^–^ → OTf^–^) produces negligible effects in protic solvents, confirming that anion coordination does not perturb the cage framework. The sole exception occurs for OTf^–^ salts in MeCN, where the short-wavelength CD band (∼210–215 nm) intensifies markedly and the UV–vis tail broadens. These high-energy perturbations, absent in polar protic media, are consistent with enhanced ion-pairing in the low-dielectric MeCN environment, where outer-sphere OTf^–^ interactions modulate the electronic structure without disrupting the overall cage geometry.

Remarkably, the core CD topology remains invariant across all conditions. For ***S*** **-1**, the characteristic negative–positive-negative pattern persists in all media, with only moderate solvatochromic shifts: the long-wavelength minimum blue-shifts slightly from ∼282 nm (MeOH) to ∼274 nm (H_2_O), while MeCN sharpens the bands and enhances the positive contribution near 246 nm. Similarly, **ZnZn** maintains its diagnostic CD fingerprint, though amplitudes vary systematically attenuated in H_2_O, enhanced at short wavelengths (∼215 nm) in MeOH, and most pronounced in MeCN where the 236–246 nm positive doublet intensifies alongside the ∼ 286 nm negative band.

Overall, the chiroptical and UV–vis signatures of ***S*** **-1** and **ZnZn** are consistent with the idea that the architecture is preserved across diverse chemical environments. Observed variations reflect small solvatochromic and ion-pairing effects rather than conformational changes, confirming that the CD evolution during metalation (Figure 2a) reports only genuine structural reorganization. However, experimental spectra alone cannot reveal the molecular origin of this reorganization. In other words, the precise geometric distortions driving the chiroptical response remain obscured. To bridge this gap, we turned to DFT and TD-DFT calculations on ***S*** **-1**, **ZnH**_**4**_, and **ZnZn**.

### DFT and TD-DFT Analysis

To establish the structural origin of the observed chiroptical evolution, we performed a systematic conformational exploration using DFT calculations on all three species ***S*** **-1**, **ZnH**_**4**_, and **ZnZn**. (see Tables S1–S7 and Section S4).^44^ The optimized lowest-energy structures reveal a progressive geometric reorganization upon stepwise metalation that crucially preserves the overall *C*_3_-symmetric propeller-like topology of the TPMA units while systematically modulating the cage architecture (Figures 3 and S29–S34 and Tables S8–S10).

**Figure 3:**
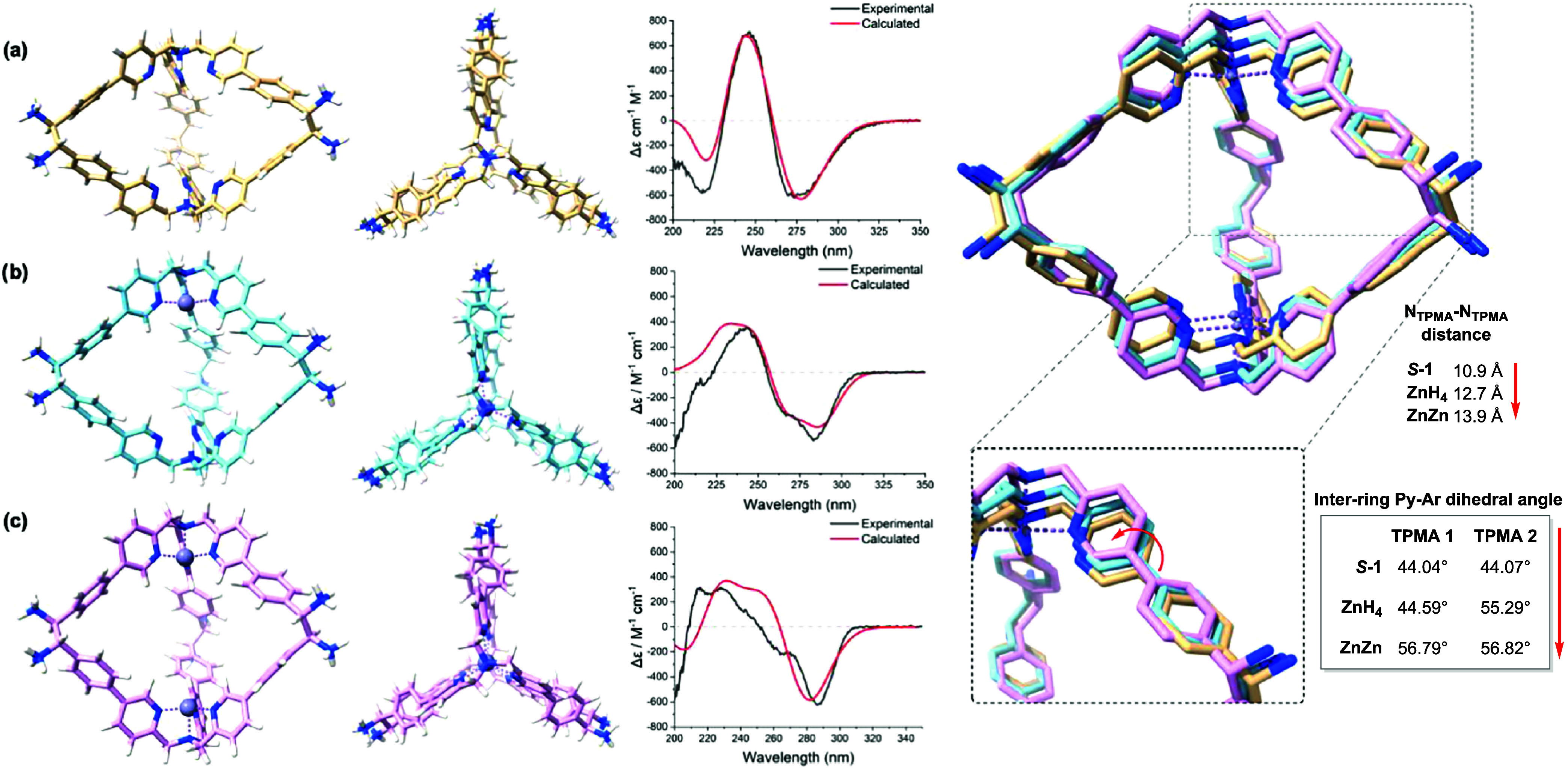
Left: Side and top views of the optimized DFT structures of the three species (a) ***S***-1, (b) **ZnH**_**4**_ and (c) **ZnZn**. Middle: comparison between experimental and computed ECD spectra of ***S***-1 (a), **ZnH**_**4**_ (b), and **ZnZn** (c). Right: Superimposed DFT-optimized structures highlighting the progressive cage expansion upon stepwise Zn­(II) incorporation, with a zoomed view of the pyridine-aryl dihedral angle (***S***-1 yellow, **ZnH**_**4**_ blue, **ZnZn** pink). Theoretical spectra were convoluted with Gaussian functions using half-width at half-height (HWHH) values of 0.333 eV (***S***-1), 0.11 eV (**ZnH**_**4**_), and 0.15 eV (**ZnZn**), selected to best reproduce the experimental bandwidths and account for the progressive conformational distortion upon metalation.

#### Local Coordination Geometry and Preorganization

**TPMA** Zn­(II)-bound pockets, one in **ZnH**_**4**_ and two in **ZnZn**, are structurally equivalent in both structures. Each Zn­(II) maintains canonical κ^4^-N tetrahedral coordination (Zn–N­(tert) ≈ 2.02 Å, Zn–N­(py) ≈ 2.06 Å), consistent with slightly distorted tetrahedral geometry typical of **TPMA** complexes.^42,43^ Within metalated pockets, the three pyridyl nitrogen atoms adopt a convergent arrangement with an average N­(py)···N­(py) distance of 3.55 Å. In contrast, unbound **TPMA** pockets exhibit systematically larger N­(py)···N­(py) separations: 4.18 Å in ***S*** **-1** (two unbound sites) and 4.00 Å in **ZnH**_**4**_ (one remaining unbound site). This progressive contraction, from 4.18 Å (***S*** **-1**) to 4.00 Å (**ZnH**_**4**_) to 3.55 Å (Zn-bound), indicates that the first metalation event preorganizes the second binding pocket, reducing the conformational penalty for subsequent Zn­(II) incorporation and providing a structural basis for the observed kinetically cooperative behavior.

#### Global Framework Reorganization

At the global level, stepwise metalation induces a coupled biphasic structural response. The inter-**TPMA** distance (measured between tertiary N atoms) increases systematically from 10.95 Å (***S*** **-1**) to 12.73 Å (**ZnH**_**4**_) to 13.98 Å (**ZnZn**), corresponding to a cumulative 3.03 Å (27%) elongation along the cage’s principal axis. Simultaneously, however, the distance between external ammonium ions on different arms contracts from 19.37 Å (***S*** **-1**) to 18.63 Å (**ZnH**_**4**_) to 18.14 Å (**ZnZn**), reflecting compression of the cage periphery. This phenomenon, axial expansion coupled with peripheral contraction, is reflected also in *intra*-arm ammonium dihedral angles, which decrease systematically from 58° (***S*** **-1**) to 53° (**ZnH**_**4**_) to 49° (**ZnZn**). In the free cage, electrostatic repulsion between protonated ammonium groups drives an “extended” cage conformation that maximizes NH_3_^+^···NH_3_^+^ separation. Upon Zn­(II) coordination, the rigid **TPMA**–metal chelate enforces a more compact arrangement, forcing the flexible ammonium-terminated linkers to adopt folded conformations that bring the charged termini closer together despite the increased **TPMA** separation.

The DFT-derived geometries reveal the molecular basis for long-range structural communication between the two **TPMA** sites (Figure 3). Metal coordination imposes a dual structural constraint: (*i*) the Zn­(II) center locks the pyridyl arms into a convergent κ^4^ tetrahedral arrangement (N­(py)···N­(py) ≈ 3.55 Å), and (ii) this rigid coordination geometry propagates through the covalent framework, enforcing a systematic reorganization that preorganizes the second **TPMA** site (4.18 → 4.00 Å N­(py) contraction) and simultaneously compresses the cage periphery (19.37 → 18.14 Å NH_3_^+^ approach). The opposing trends, **TPMA** divergence vs. ammonium convergence, arise because the unbound cage conformation is dictated primarily by electrostatic repulsion between charged termini, whereas the metalated structure is governed by the geometric requirements of the **TPMA** Zn­(II) chelate. The solvent dependence of the *k*_2_/*k*_1_ ratio (lower in MeCN/H_2_O than in pure H_2_O) is consistent with this model: in high-polarity media, the electrostatic penalty of bringing ammonium groups closer together is lowered, increasing the rate of the second metalation step and amplifying the kinetic differentiation between the two coordination events.

The excellent agreement between experimental and TD-DFT-calculated CD spectra (B3LYP/6–31G­(d), PCM-MeCN, 120 singlets)^45^ validates the structural accuracy of the DFT-optimized geometries (See Section S5 and Figures S35–S37). Theoretical ECD spectra were convoluted for ***S*** **-1**, **ZnH**_**4**_, and **ZnZn**, respectively, chosen to best reproduce the experimental bandwidths and reflect an apparent reduction in conformational freedom along the series (Figure 3). For consistency, theoretical traces were scaled uniformly per species to match experimental amplitudes.

For ***S*** **-1**, the calculated spectrum correctly reproduces the bisignate topology and the positions of the main features (calc. 244–278 nm vs exp. 246–273 nm). For **ZnH**_**4**_, the simulation resolves the two negative contributions at ∼285 nm and ∼270 nm, the latter appearing experimentally as a shoulder, as well as the positive band with its characteristic shoulder in the 220–240 nm region. The negative far-UV contribution observed experimentally in the ∼200–215 nm is underestimated, likely due to the finite excited-state window and the strong solvation dependence of these high-energy transitions.

For **ZnZn**, TD-DFT reproduces the long-wavelength negative band (282–287 nm) and the short-wavelength pair of positive bands, although with a slight blue-shift and enhanced separation compared to experiment. The experimentally observed shoulder at ∼265–270 nm is not fully resolved theoretically. Overall, the simulations accurately display blue-shift and redistribution of intensities occurring along the sequence ***S*** **-1** → **ZnH4** → **ZnZn**, supporting the view that Zn­(II) coordination progressively modifies the relative orientation and electronic coupling of the **TPMA** chromophores without altering the underlying chiral topology.

The combined experimental and computational data provide a coherent picture of how stepwise Zn­(II) coordination governs both the structural and chiroptical evolution of the cage. NMR kinetic experiments reveal a clear sequential process, in which the first coordination event proceeds rapidly, whereas the second becomes sensitive to medium composition.

## Conclusion

Stepwise Zn­(II) coordination in symmetric **TPMA**-cage ***S*** **-1** exhibits apparent positive cooperativity (*k*_2_/*k*_1_ ≈ 1.5), consistent with a kinetically cooperative metalation process and associated with allosteric-like structural communication across >10 Å. While NMR kinetics provide evidence for sequential metalation, circular dichroism (CD) spectroscopy proves indispensable for revealing the underlying structural mechanism. Five isodichroic points track the discrete ***S*** **-1** → **ZnH**_**4**_ → **ZnZn** progression with exceptional clarity, enabling detection of conformational changes invisible to NMR. DFT calculations quantify the CD-revealed mechanism: initial Zn­(II) binding contracts the unbound TPMA pocket (N···N: 4.18 → 4.00 Å) while triggering compensatory peripheral adjustments, reducing the conformational penalty for subsequent coordination despite electrostatic penalties. TD-DFT validation establishes quantitative correlation between chiroptical signatures and geometric reorganization, confirming that CD serves as a direct structural reporter of metalation events in chiral cages.

This work demonstrates that CD spectroscopy, when combined with computational validation, provides access to structural information not readily available through conventional methods, establishing it as an essential tool for investigating cooperative mechanisms in symmetric supramolecular systems. The ability to track conformational evolution throughout multistep coordination processes opens new avenues for characterizing structural communication processes in synthetic architectures, including allosteric-type responses, with implications for rational design of heterometallic catalysis, responsive materials, and functional assemblies where structural communication governs reactivity.

## Experimental Section

### Materials and Methods

Cage ***S*** **-1** was synthesized according to literature.^28^ All reagents and solvents were obtained from commercial suppliers and used as received unless otherwise stated. Solutions were prepared using deionized water and spectroscopic-grade solvents. Details regarding sample preparation, NMR titration experiments, and spectroscopic measurements are provided in the Supporting Information.

#### Spectroscopic Measurements

^**1**^H NMR spectra were recorded at 298 K in D_2_O or CD_3_CN/D_2_O mixtures. Kinetic experiments were performed by monitoring the evolution of characteristic signals upon incremental addition of Zn­(II) salts. UV–vis and circular dichroism (CD) spectra were recorded under identical conditions, and titrations were carried out by stepwise addition of ZnCl_2_ or Zn­(OTf)_2_ to solutions of ***S*** **-1**. Full experimental procedures and data analysis methods are described in the Supporting Information.

#### Computational Methods

Density functional theory (DFT) and time-dependent DFT (TD-DFT) calculations were performed using Gaussian 16.^44^ Geometry optimizations and excited-state calculations were carried out using the B3LYP functional and 6–31G­(d) basis set, with implicit solvation (PCM, MeCN). Further details on conformational searches, computational protocols, and spectral simulations are provided in the Supporting Information.

## Supplementary Material



## Notes

Experimental details; NMR kinetic experiments and traces; CD measurements and the detailed conformational search among the structures (DFT, TF-DFT calculations) (PDF)
