# Supplementary material for: Circular Dichroism Reveals Positive Cooperativity through Compensatory Motion in Stepwise Cage Metalation
Source: Inorg Chem. 2026 Mar 31;65(14):8174–81. doi: 10.1021/acs.inorgchem.6c00988 (PMC13080976; doi:10.1021/acs.inorgchem.6c00988)
Supplement: Supplementary file 1 [file ic6c00988_si_001.pdf]

# Supporting Information

# Circular Dichroism Reveals Positive Cooperativity through Compensatory Motion in Stepwise Cage Metalation

Melvin Raulin,[*,a,b,c] Chiara Slaviero,[a] Federico Begato,[a] Giulia Licini,[a,b] and Cristiano Zonta[*,a,b]

a. Department of Chemical Sciences, University of Padova, via F. Marzolo 1, 35131, Padova (Italy).

b. CIRCC - Consorzio Interuniversitario per le Reattività Chimiche e la Catalisi, via C. Ulpiani 27, 70126, Bari (Italy).

c. Aix Marseille Univ, CNRS, Centrale Med, ISM2, 13013 Marseille (France).

E-mails: cristiano.zonta@unipd.it, melvin.raulin@univ-amu.fr

# Table of Content

1. Materials and Methods ..... 3
2. $^1$H NMR kinetic studies ..... 3
2.1. *S*-1 with 0.5 equiv. $Zn(OTf)_2$ in $D_2O$ ..... 4
2.2. *S*-1 with 1.0 equiv. $Zn(OTf)_2$ in $D_2O$ ..... 5
2.3. *S*-1 with 2.0 equiv. $Zn(OTf)_2$ in $D_2O$ ..... 6
2.4. *S*-1 with 0.2 equiv. $Zn(OTf)_2$ in $CD_3CN/D_2O$ (1:1) ..... 7
2.5. *S*-1 with 0.4 equiv. $Zn(OTf)_2$ in $CD_3CN/D_2O$ (1:1) ..... 8
2.6. *S*-1 with 0.5 equiv. $Zn(OTf)_2$ in $CD_3CN/D_2O$ (1:1) ..... 9
2.7. *S*-1 with 1.0 equiv. $Zn(OTf)_2$ in $CD_3CN/D_2O$ (1:1) ..... 10
2.8. *S*-1 with 2.0 equiv. $Zn(OTf)_2$ in $CD_3CN/D_2O$ (1:1) ..... 11
2.9. Time-resolved kinetic profiles derived from NMR integration ..... 12
2.10. Kinetic data elaboration ..... 15
3. CD Measurements ..... 17
4. Computational Section ..... 23
4.1 Conformational analysis ..... 23
4.2. Energy Calculation of the most stable conformers ..... 25
4.3. B3LYP/6-31G(d) Minimized Structures ..... 27
4.4. Structural Parameters of the Lowest-Energy Conformations ..... 29
5. TD Calculations ..... 30
6. DFT minimized geometry coordinates ..... 32
6.1 Cage *S*-1 B ..... 32
6.2 Cage $ZnH_4$ B ..... 34
6.3 Cage ZnZn B ..... 37
References ..... 40

# 1. Materials and Methods

All reagents were obtained from standard suppliers and utilized without additional purification. ***S*-1** and **ZnZn** were synthesized following literature procedures.[S1] Chemicals were purchased from Merck, TCI, or Apollo Scientific and used without further purification. NMR spectra were recorded at 298 K on Bruker Avance-300 MHz and Bruker 400 Avance III BBi-z grad 5 mm. The NMR data were processed using Bruker Topspin 3.5 pl2 and MestReNova 12.0.0. Kinetic NMR data were processed using DynaFit 4 (http://www.biokin.com/dynafit/index.html). ECD spectra were recorded with a Jasco J-1500 spectrometer and processed with Spectra Manager Version 2.15.3.1 or OriginPro 2018 (64-bit) SR1 b9.5.1.195. The computational searches of the most stable structures and the TD-DFT calculations were carried out Gaussian 16 package1 Revision C.01 and processed with GaussView 6.0.162 or CYL view BETA 1.0. The experimental/theoretical comparison of the CD spectra were carried out with SpecDis Version 1.713.

# 2. $^1$H NMR kinetic studies

In a 5 mm NMR tube was introduced a solution of cage ***S*-1** (0.5 mM, 500 µL) in $D_2O$ or $CD_3CN/D_2O$ (1:1, v/v). A reference $^1$H NMR spectrum of the cage solution was first recorded prior to metal addition (t = 0). Then, a defined amount of a $ZnCl_2$ or $Zn(OTf)_2$ stock solution (prepared in the same solvent) was then added to deliver the desired equivalents (0.2-2.0 equiv., final volume < 10%). The tube was capped and mixed, after which the time-resolved acquisition was started. Consecutive $^1$H spectra were acquired every 10 min for a total of 60 spectra.

## 2.1. *S*-1 with 0.5 equiv. $Zn(OTf)_2$ in $D_2O$

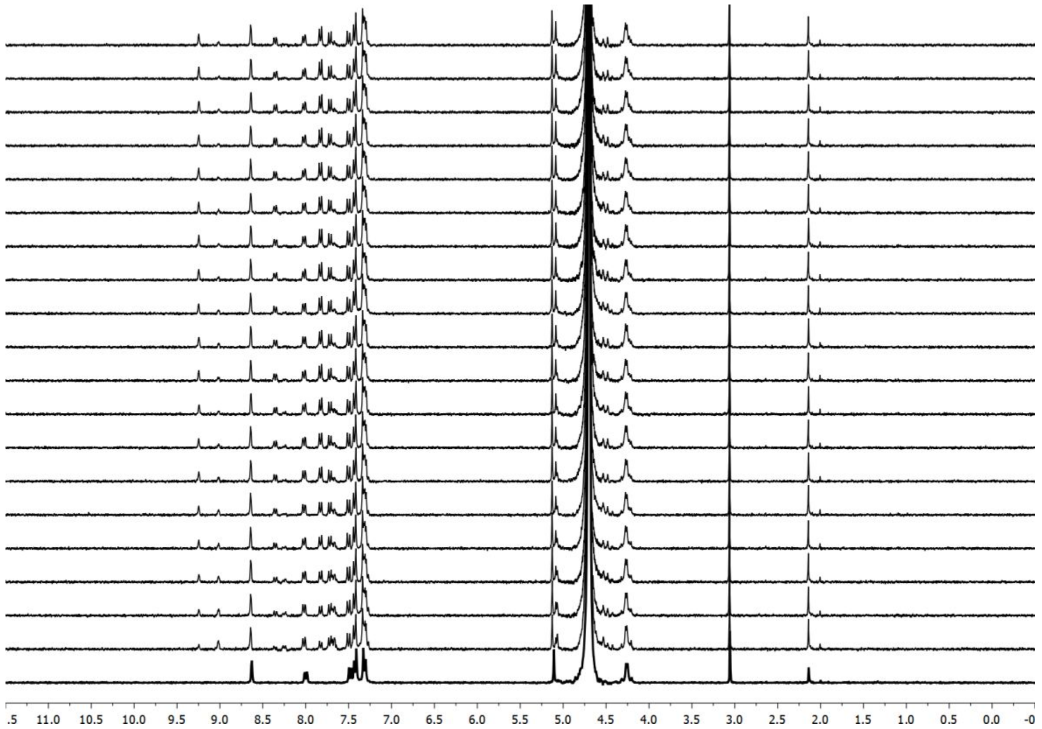


**Figure S1.** $^{1}$H-NMR kinetic study of Cage ***S*-1** (0.5 mM, with $OTf^-$ as counterions) upon addition of 0.5 equivalent of $Zn(OTf)_2$ in $D_2O$ (300 MHz, 298 K).

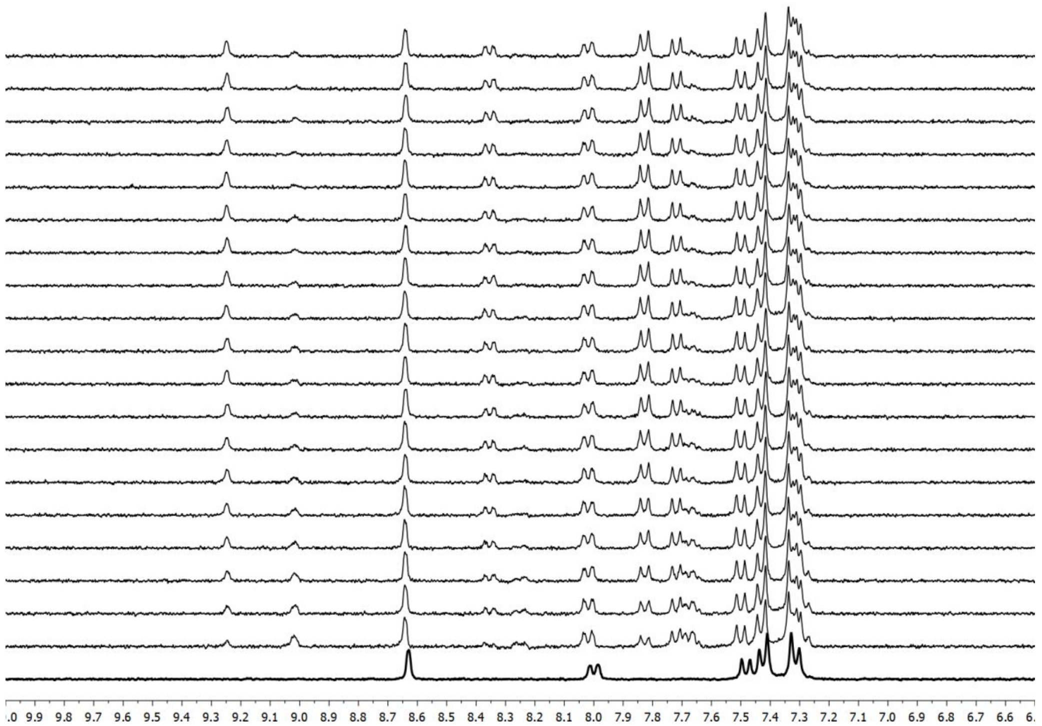


**Figure S2.** Partial $^{1}$H-NMR kinetic study of Cage ***S*-1** (0.5 mM, with $OTf^-$ as counterions) upon addition of 0.5 equivalent of $Zn(OTf)_2$ in $D_2O$ (300 MHz, 298 K).

## 2.2. *S*-1 with 1.0 equiv. $Zn(OTf)_2$ in $D_2O$

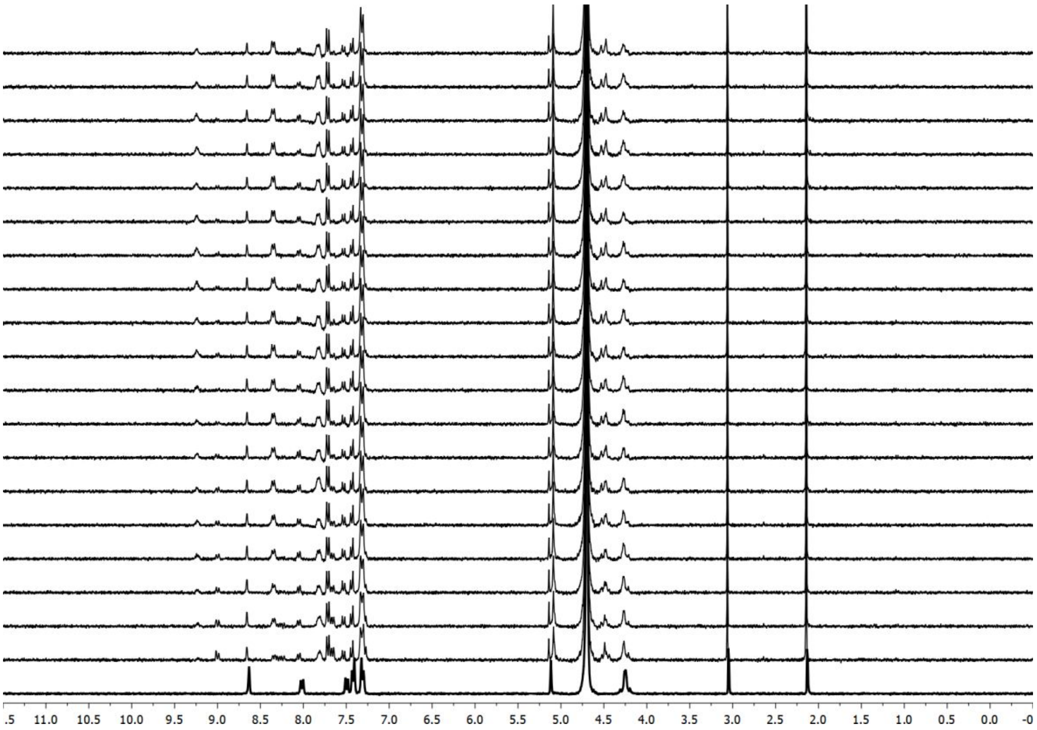


**Figure S3.** $^{1}$H-NMR kinetic study of Cage ***S*-1** (0.5 mM, with $OTf^{-}$ as counterions) upon addition of 1.0 equivalent of $Zn(OTf)_2$ in $D_2O$ (300 MHz, 298 K).

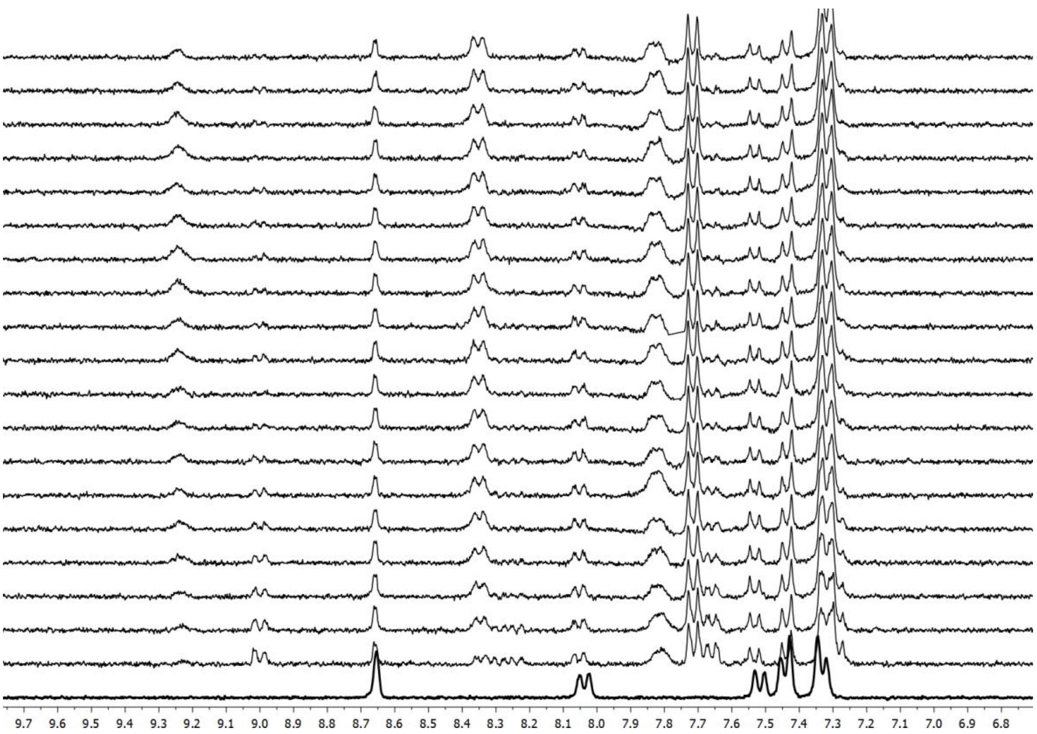


**Figure S4.** Partial $^{1}$H-NMR kinetic study of Cage ***S*-1** (0.5 mM, with $OTf^{-}$ as counterions) upon addition of 1.0 equivalent of $Zn(OTf)_2$ in $D_2O$ (300 MHz, 298 K).

## 2.3. *S*-1 with 2.0 equiv. $Zn(OTf)_2$ in $D_2O$

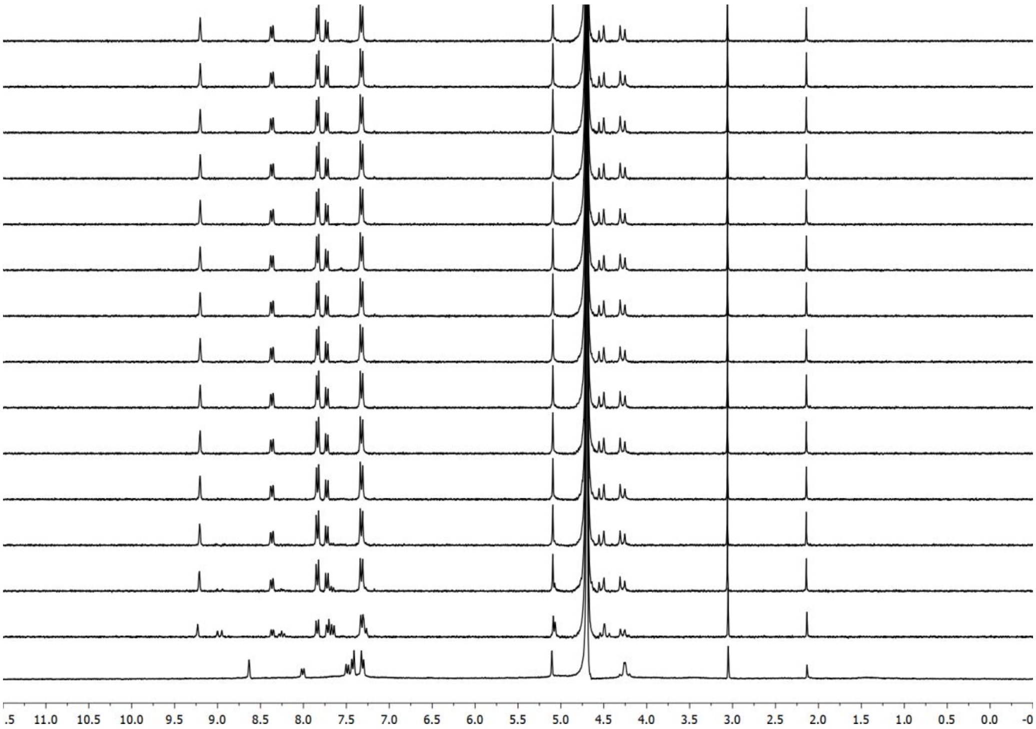


**Figure S5.** $^{1}$H-NMR kinetic study of Cage ***S*-1** (0.5 mM, with $OTf^-$ as counterions) upon addition of 2.0 equivalents of $Zn(OTf)_2$ in $D_2O$ (300 MHz, 298 K).

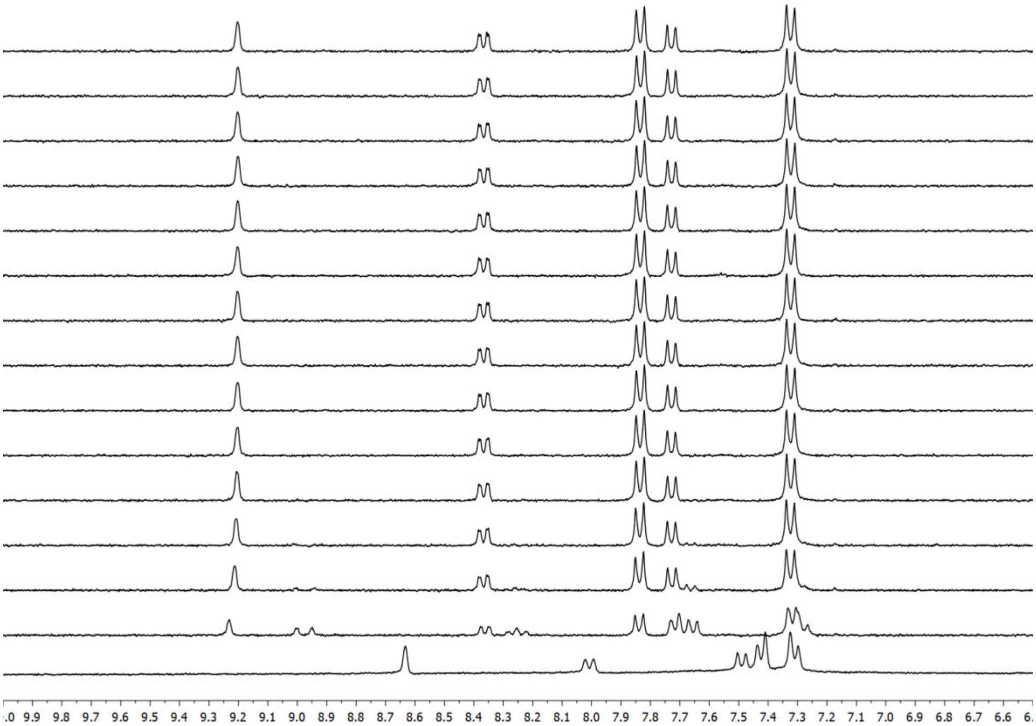


**Figure S6.** Partial $^{1}$H-NMR kinetic study of Cage ***S*-1** (0.5 mM, with $OTf^-$ as counterions) upon addition of 2.0 equivalents of $Zn(OTf)_2$ in $D_2O$ (300 MHz, 298 K).

## 2.4. *S*-1 with 0.2 equiv. $Zn(OTf)_2$ in $CD_3CN/D_2O$ (1:1)

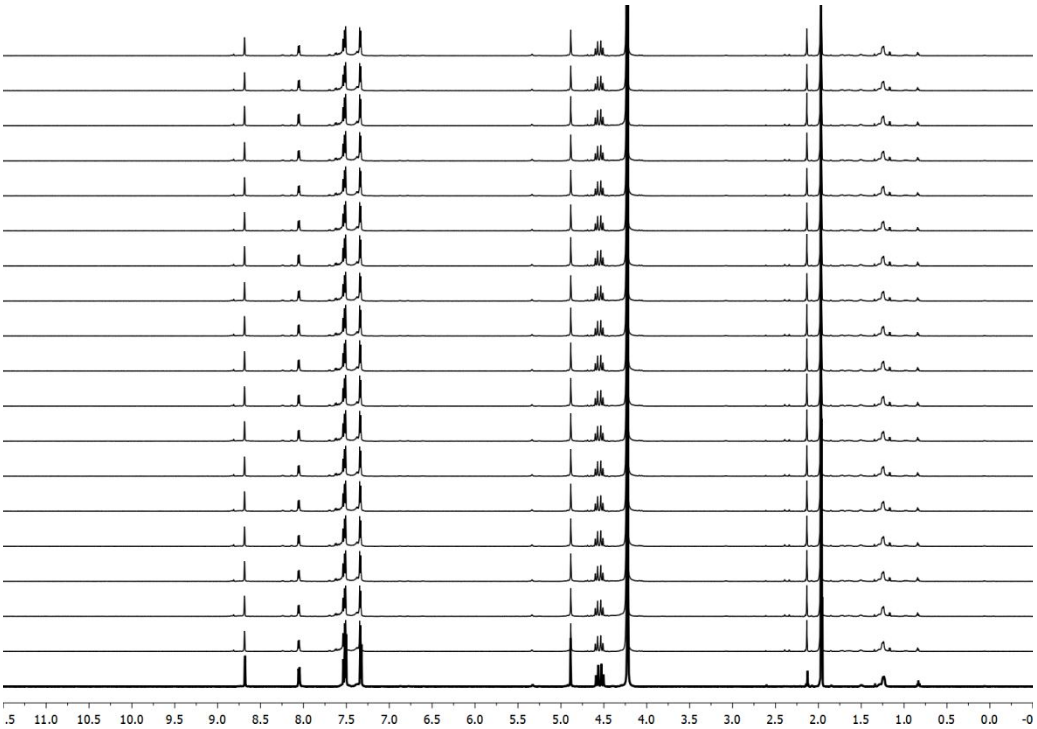


**Figure S7.** $^{1}$H-NMR kinetic study of Cage ***S*-1** (0.5 mM, with $OTf^-$ as counterions) upon addition of 0.2 equivalent of $Zn(OTf)_2$ in $CD_3CN/D_2O$ 1:1 (300 MHz, 298 K).

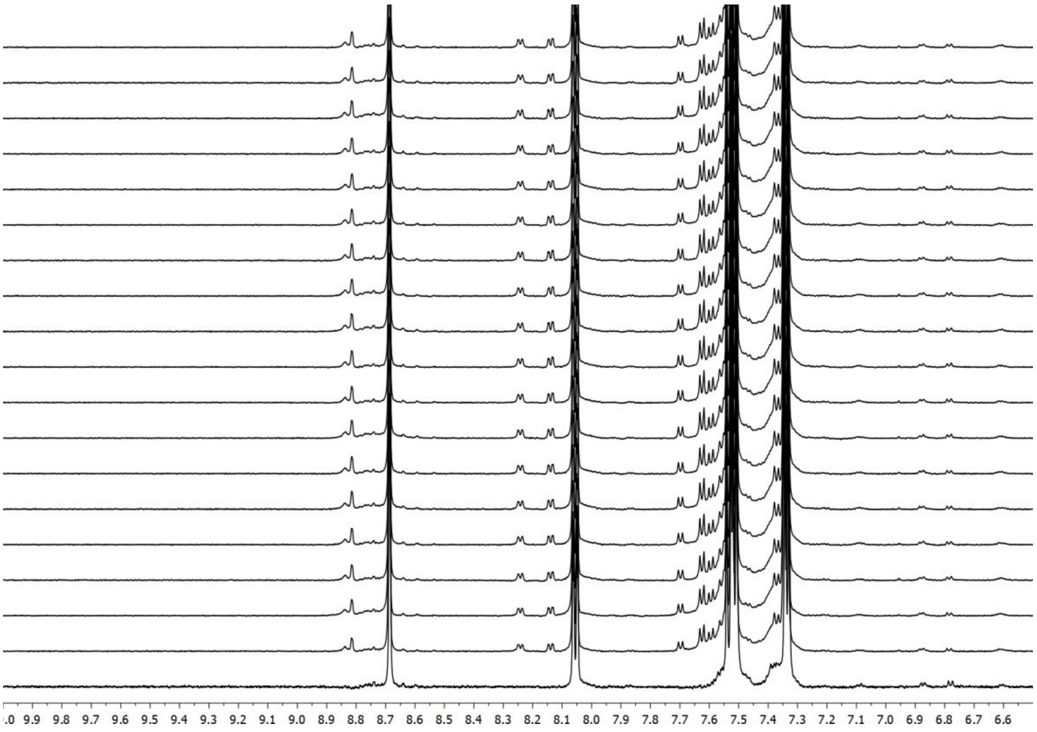


**Figure S8.** Partial $^{1}$H-NMR kinetic study of Cage ***S*-1** (0.5 mM, with $OTf^-$ as counterions) upon addition of 0.2 equivalent of $Zn(OTf)_2$ in $CD_3CN/D_2O$ 1:1 (300 MHz, 298 K).

## 2.5. *S*-1 with 0.4 equiv. $Zn(OTf)_2$ in $CD_3CN/D_2O$ (1:1)

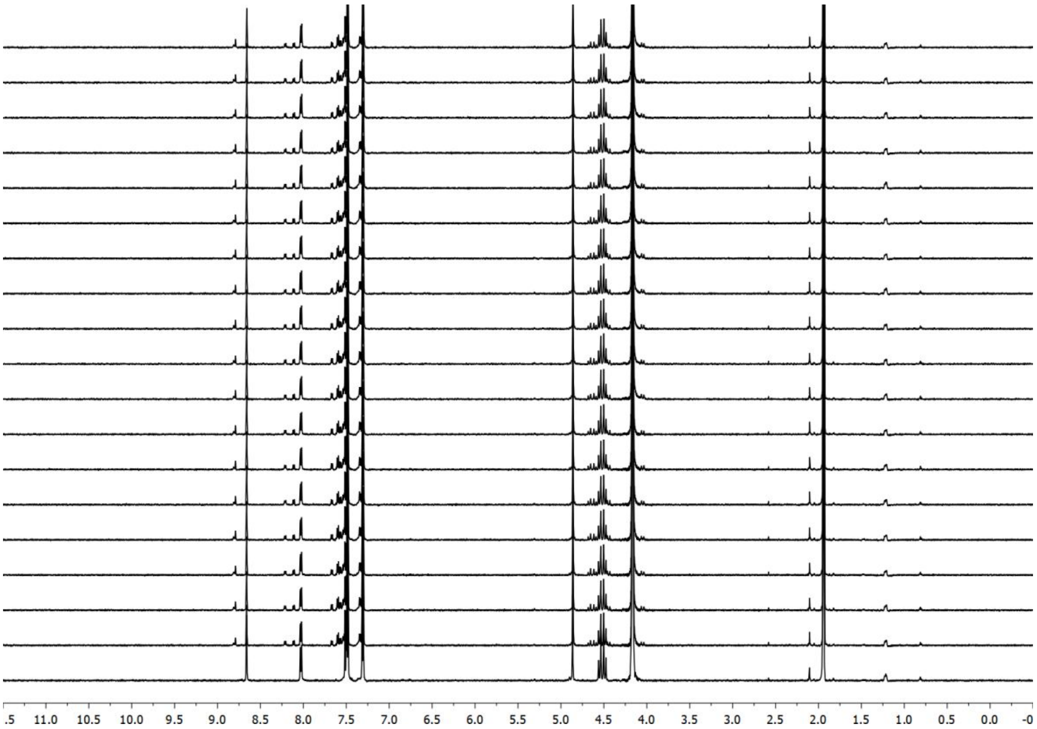


**Figure S9.** $^{1}$H-NMR kinetic study of Cage ***S*-1** (0.5 mM, with $OTf^-$ as counterions) upon addition of 0.4 equivalent of $Zn(OTf)_2$ in $CD_3CN/D_2O$ 1:1 (300 MHz, 298 K).

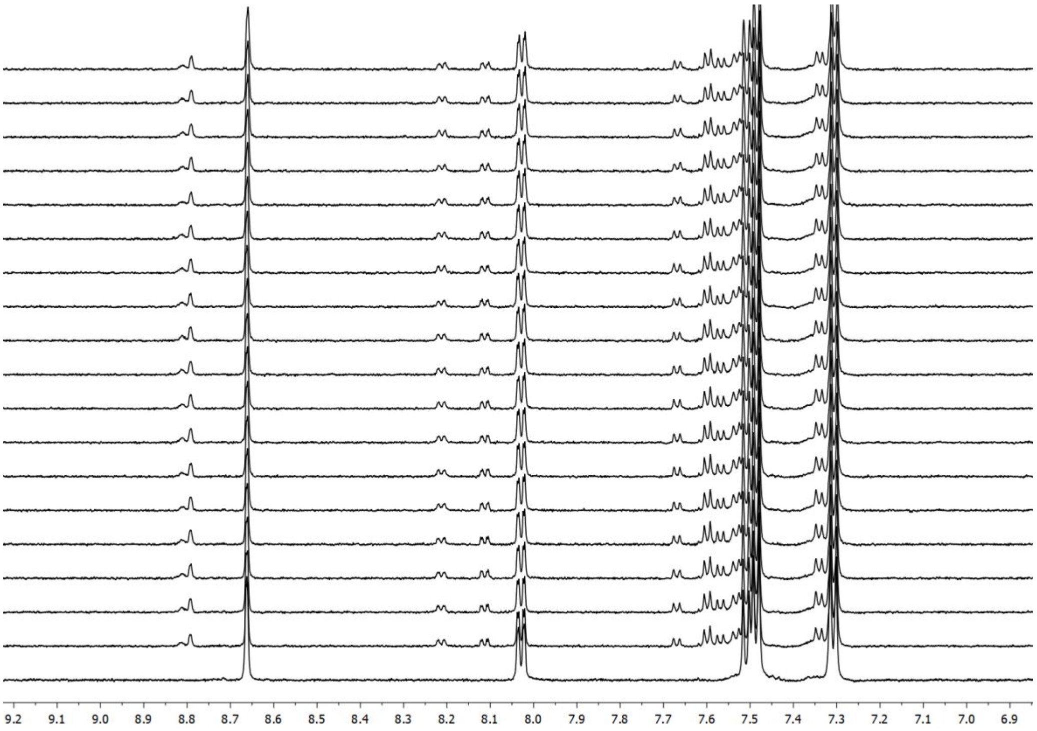


**Figure S10.** Partial $^{1}$H-NMR kinetic study of Cage ***S*-1** (0.5 mM, with $OTf^-$ as counterions) upon addition of 0.4 equivalent of $Zn(OTf)_2$ in $CD_3CN/D_2O$ 1:1 (300 MHz, 298 K).

## 2.6. *S*-1 with 0.5 equiv. $Zn(OTf)_2$ in $CD_3CN/D_2O$ (1:1)

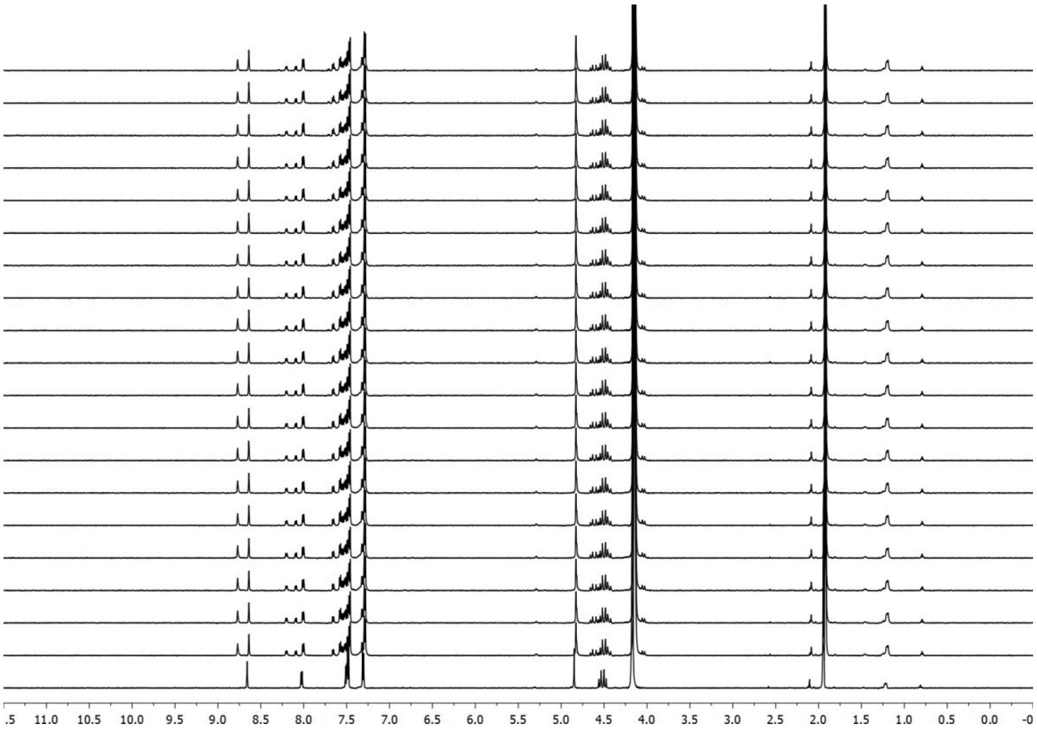


**Figure S11.** $^1$H-NMR kinetic study of Cage ***S*-1** (0.5 mM, with $OTf^-$ as counterions) upon addition of 0.5 equivalent of $Zn(OTf)_2$ in $CD_3CN/D_2O$ 1:1 (300 MHz, 298 K).

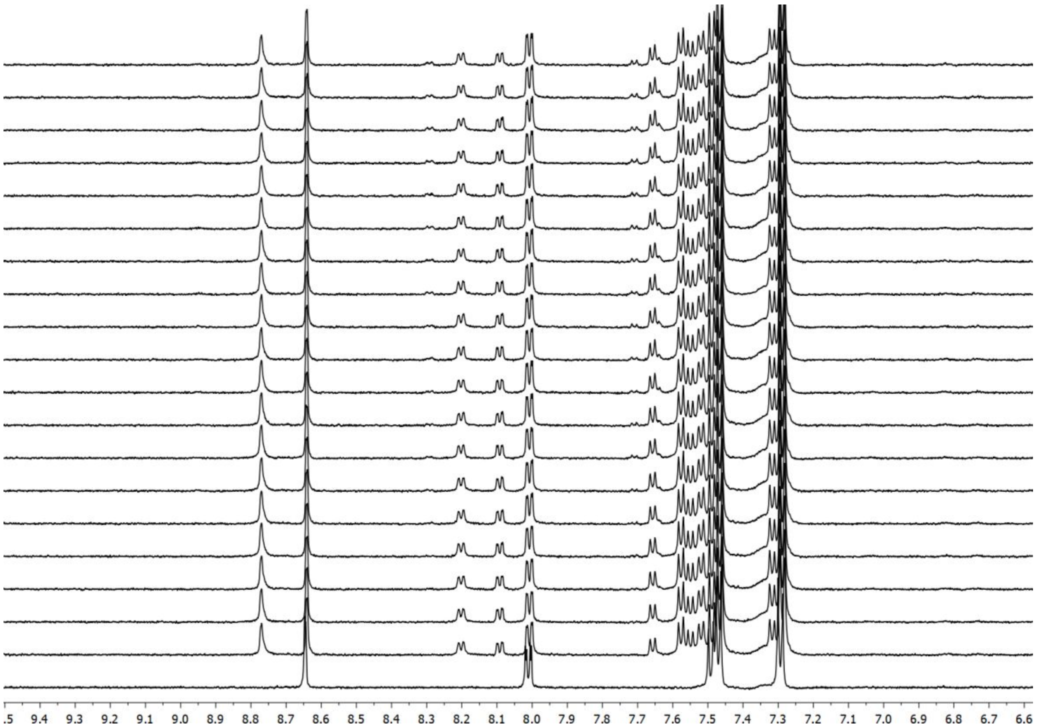


**Figure S12.** Partial $^1$H-NMR kinetic study of Cage ***S*-1** (0.5 mM, with $OTf^-$ as counterions) upon addition of 0.5 equivalent of $Zn(OTf)_2$ in $CD_3CN/D_2O$ 1:1 (300 MHz, 298 K).

## 2.7. *S*-1 with 1.0 equiv. $Zn(OTf)_2$ in $CD_3CN/D_2O$ (1:1)

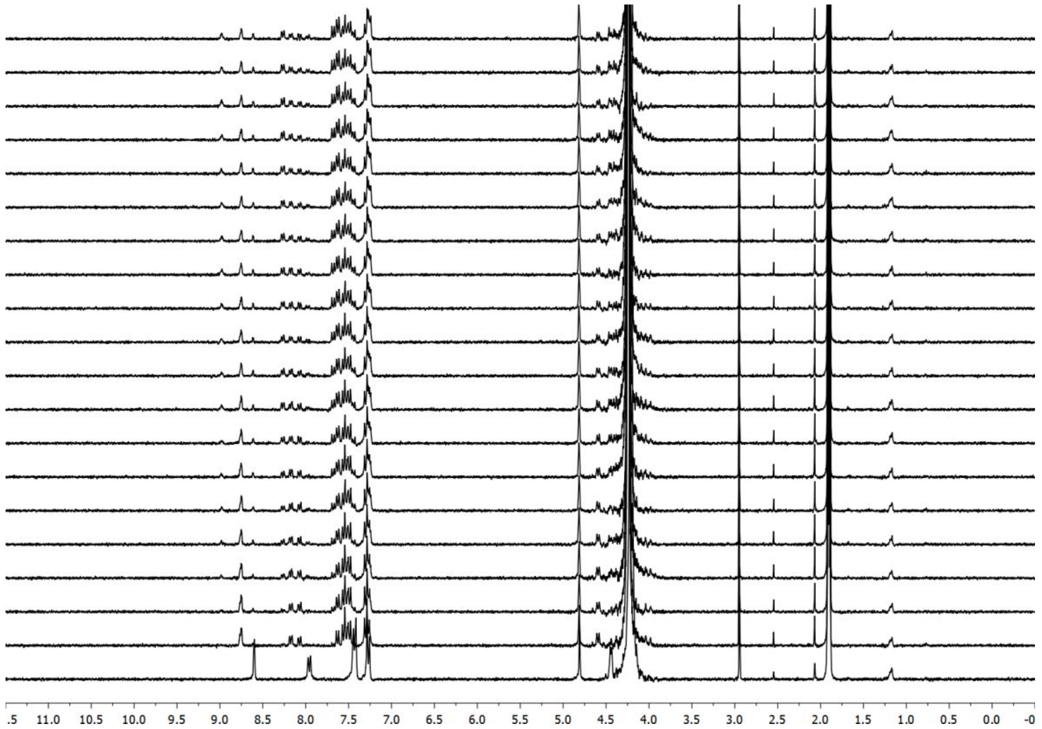


**Figure S13.** $^{1}$H-NMR kinetic study of Cage ***S*-1** (0.5 mM, with $OTf^-$ as counterions) upon addition of 1.0 equivalent of $Zn(OTf)_2$ in $CD_3CN/D_2O$ 1:1 (300 MHz, 298 K).

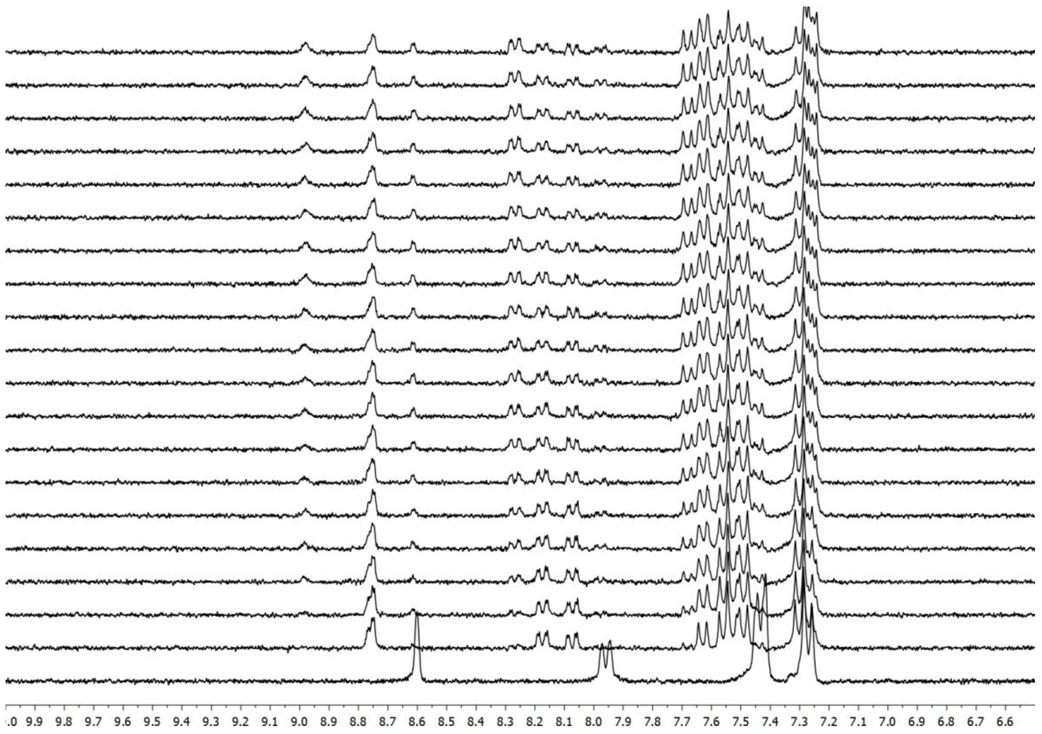


**Figure S14.** Partial $^{1}$H-NMR kinetic study of Cage ***S*-1** (0.5 mM, with $OTf^-$ as counterions) upon addition of 1.0 equivalent of $Zn(OTf)_2$ in $CD_3CN/D_2O$ 1:1 (300 MHz, 298 K).

## 2.8. *S*-1 with 2.0 equiv. $Zn(OTf)_2$ in $CD_3CN/D_2O$ (1:1)

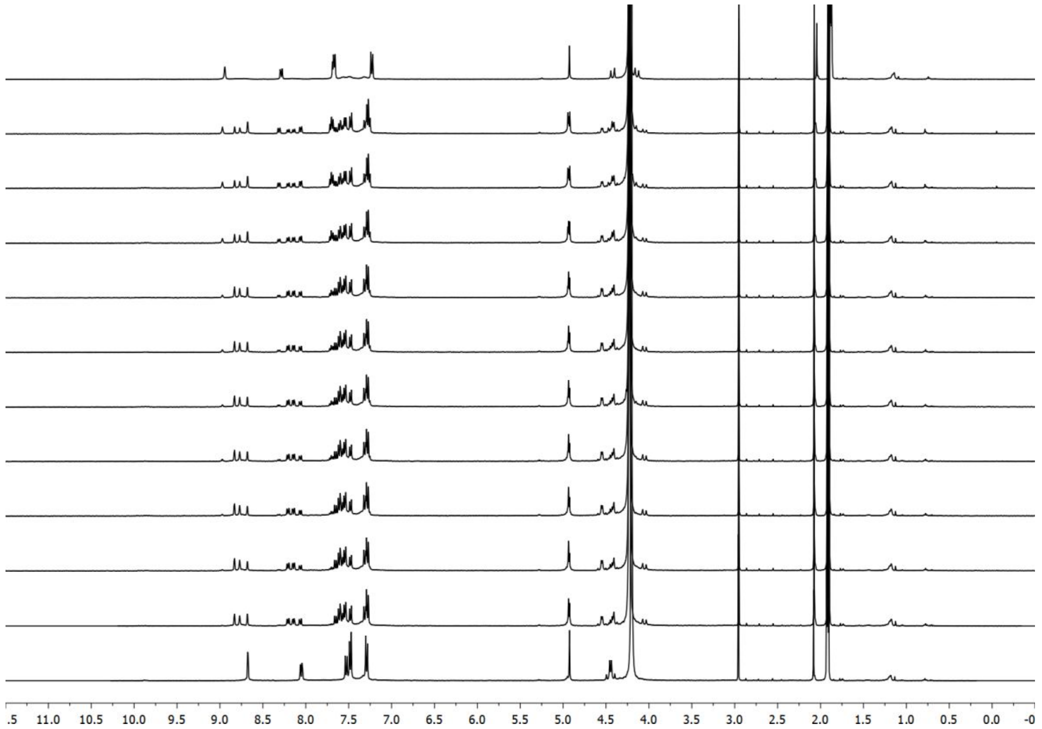


**Figure S15.** $^{1}$H-NMR kinetic study of Cage ***S*-1** (0.5 mM, with $OTf^-$ as counterions) upon addition of 2.0 equivalent of $Zn(OTf)_2$ in $CD_3CN/D_2O$ 1:1 (300 MHz, 298 K).

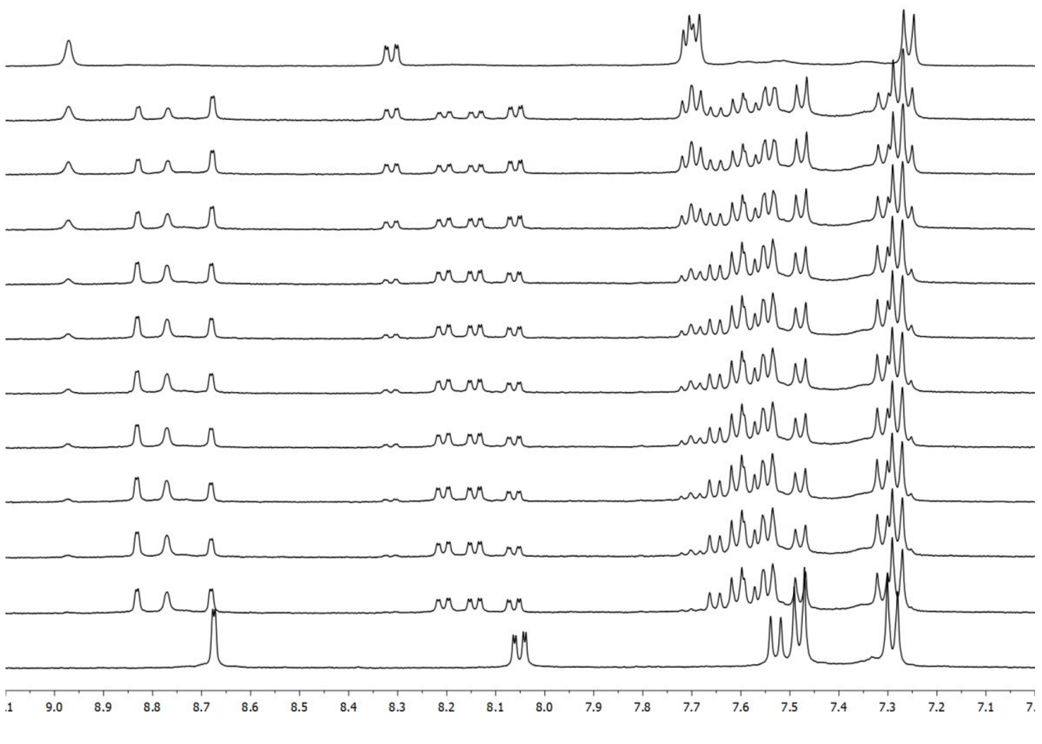


**Figure S16.** Partial $^{1}$H-NMR kinetic study of Cage ***S*-1** (0.5 mM, with $OTf^-$ as counterions) upon addition of 2.0 equivalent of $Zn(OTf)_2$ in $CD_3CN/D_2O$ 1:1 (300 MHz, 298 K).

## 2.9. Time-resolved kinetic profiles derived from NMR integration

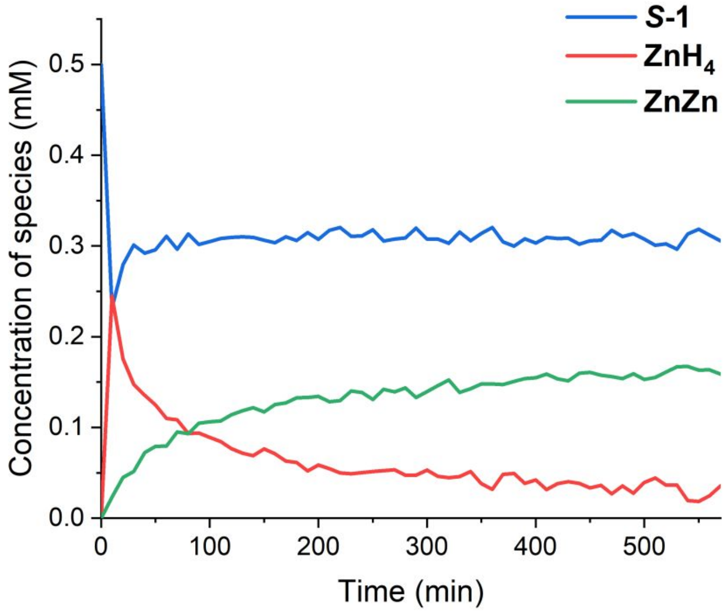


**Figure S17.** Kinetic profile for the metalation of cage ***S*-1** in $D_2O$ with 0.5 equiv. of $Zn(OTf)_2$.

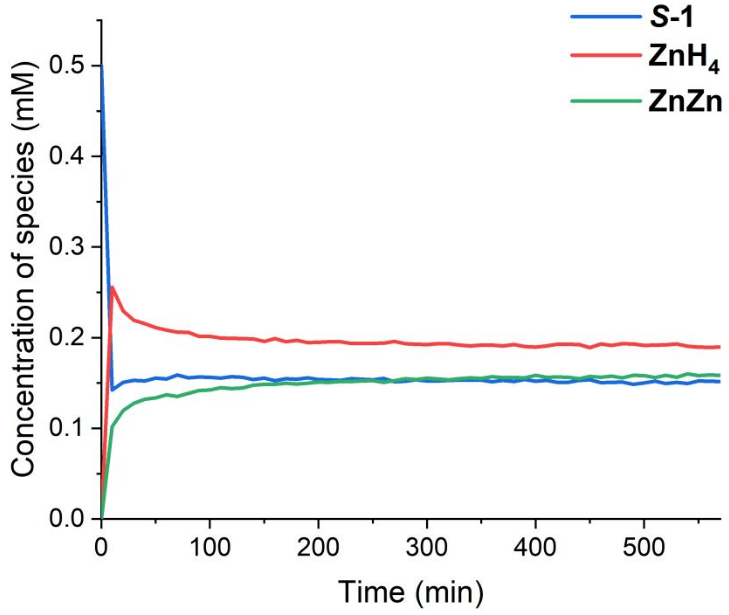


**Figure S18.** Kinetic profile for the metalation of cage ***S*-1** in $D_2O$ with 1.0 equivalent of $Zn(OTf)_2$.

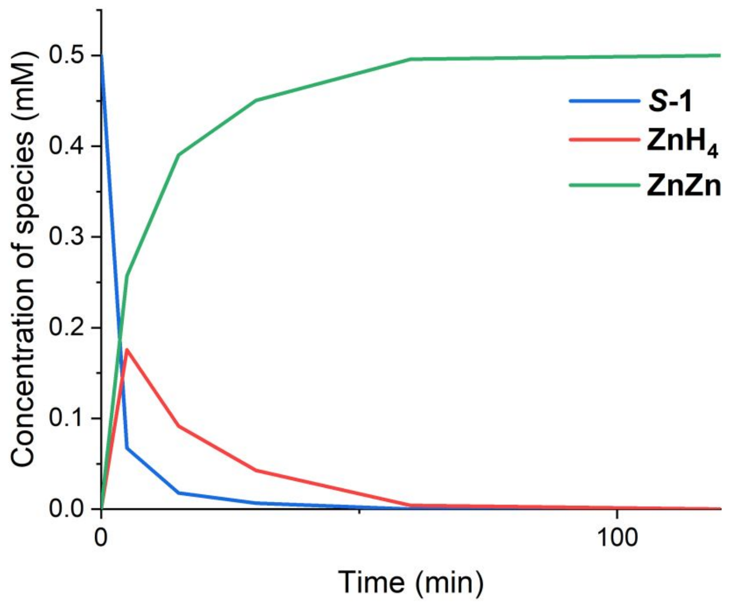


**Figure S19.** Kinetic profile for the metalation of cage ***S*-1** in $D_2O$ with 2.0 equivalents of $Zn(OTf)_2$.

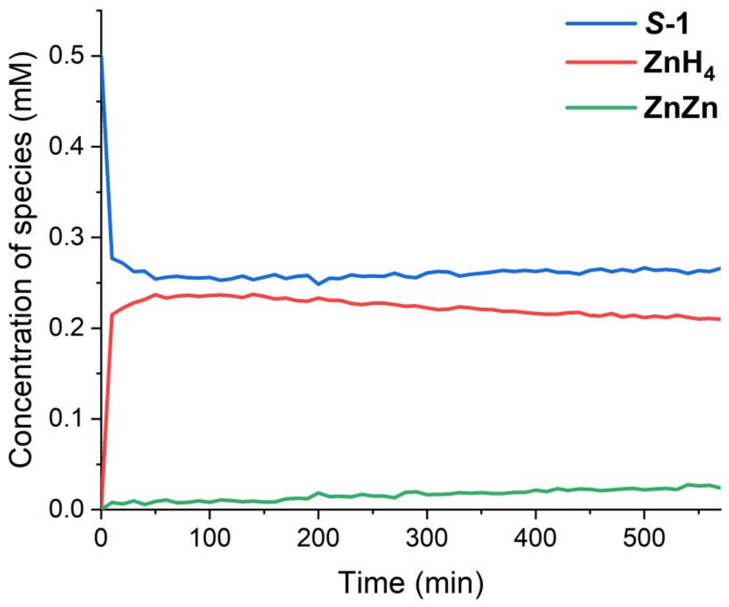


**Figure S20.** Kinetic profile for the metalation of cage ***S*-1** in $CD_3CN/D_2O$ (1:1) with 0.5 equivalents of $Zn(OTf)_2$.

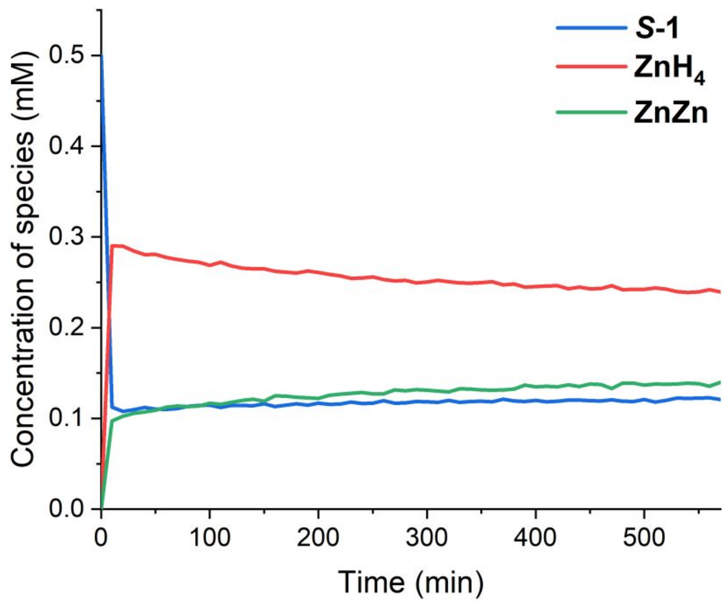


**Figure S21.** Kinetic profile for the metalation of cage ***S*-1** in $CD_3CN/D_2O$ (1:1) with 1.0 equivalent of $Zn(OTf)_2$.

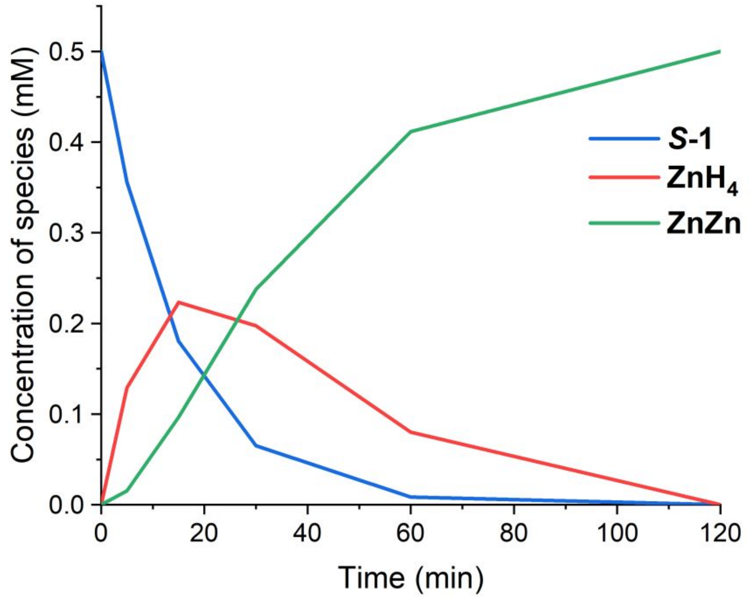


**Figure S22.** Kinetic profile for the metalation of cage ***S*-1** in $CD_3CN/D_2O$ (1:1) with 2.0 equivalents of $Zn(OTf)_2$.

## 2.10. Kinetic data elaboration

Kinetic data for the $^1$H NMR experiments were elaborated using DynaFit 4 software,[S2] which characterizes the reacting system in terms of stoichiometric equations instead of mathematical notation; the chemical equations are then translated into the underlying mathematical equations using matrix theory. DynaFit is available free of charge at http://www.biokin.com/dynafit/. The experimental plots for the consumption of ***S-1*·OTf** and the formation of species **ZnH$_4$** and **ZnZn** were fitted with a non-linear least-squares regression algorithm, according to a stepwise mechanism involving second order reactions:

(1) ***S*-1·OTf** + Zn(OTf)$_2$ → **ZnH$_4$**
(2) **ZnH$_4$** + Zn(OTf)$_2$ → **ZnZn**

The error reported for each kinetic constant value is the standard error of the fitting.

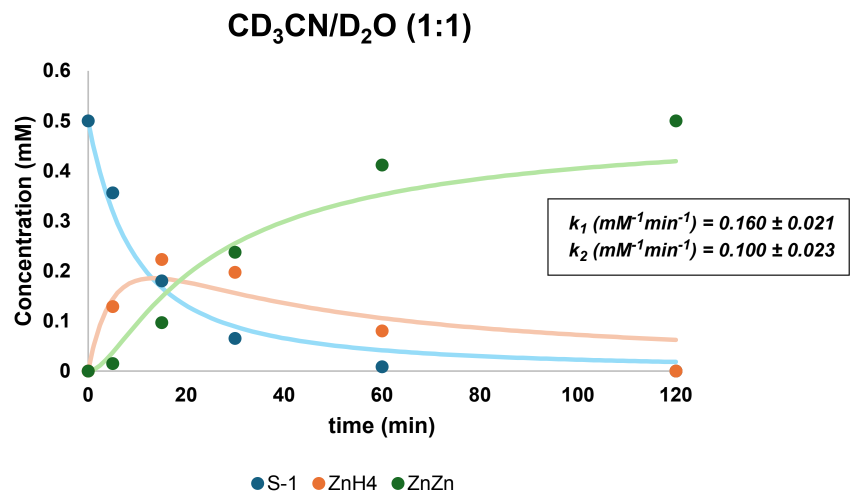


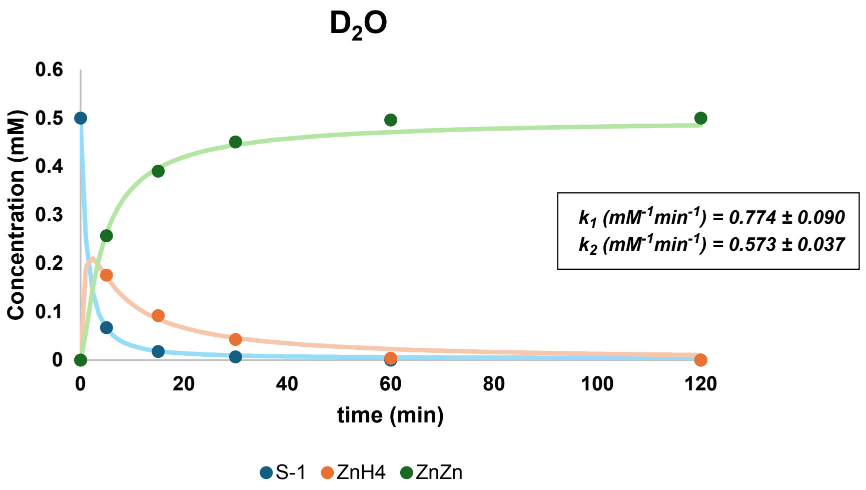


**Figure S20.** Changes in concentration of the species ***S*-1·OTf, $ZnH_4$** and **ZnZn** with time in the $CD_3CN/D_2O$ mixture (top) and in $D_2O$ (bottom) upon the addition of 2 eq. of $Zn(OTf)_2$. Solid lines represent the fit of the experimental data to the theoretical model; $k_1$ and $k_2$ are the rate constants obtained from the fitting for the first and the second step, respectively. Initial guesses for the fitting were estimated by applying initial rate method to kinetic experiments conducted at different $Zn(OTf)_2$ loadings.

## 3. CD Measurements

CD measurements were performed with solution of cage ***S*-1** in a solvent (mixture of MeCN/$H_2O$ (1:1) for the titration; acetonitrile, water or methanol for the comparative chiroptical analysis) to obtain a final concentration equal to $1.0 \cdot 10^{-4}$ M (0.1 cm cuvette). The CD spectra were measured in millidegrees, normalized for the concentration of the cage and reported as Δε, following the formula

$$\Delta\varepsilon = \frac{\Theta}{32980 \cdot C \cdot l}$$

where Δε is the molar circular dichroism (in $M^{-1}$ $cm^{-1}$) , Θ is the CD value registered from the instrument (expressed in mdeg), C is the concentration of the sample (expressed in mol/L) and l is the optical path (expressed in cm).

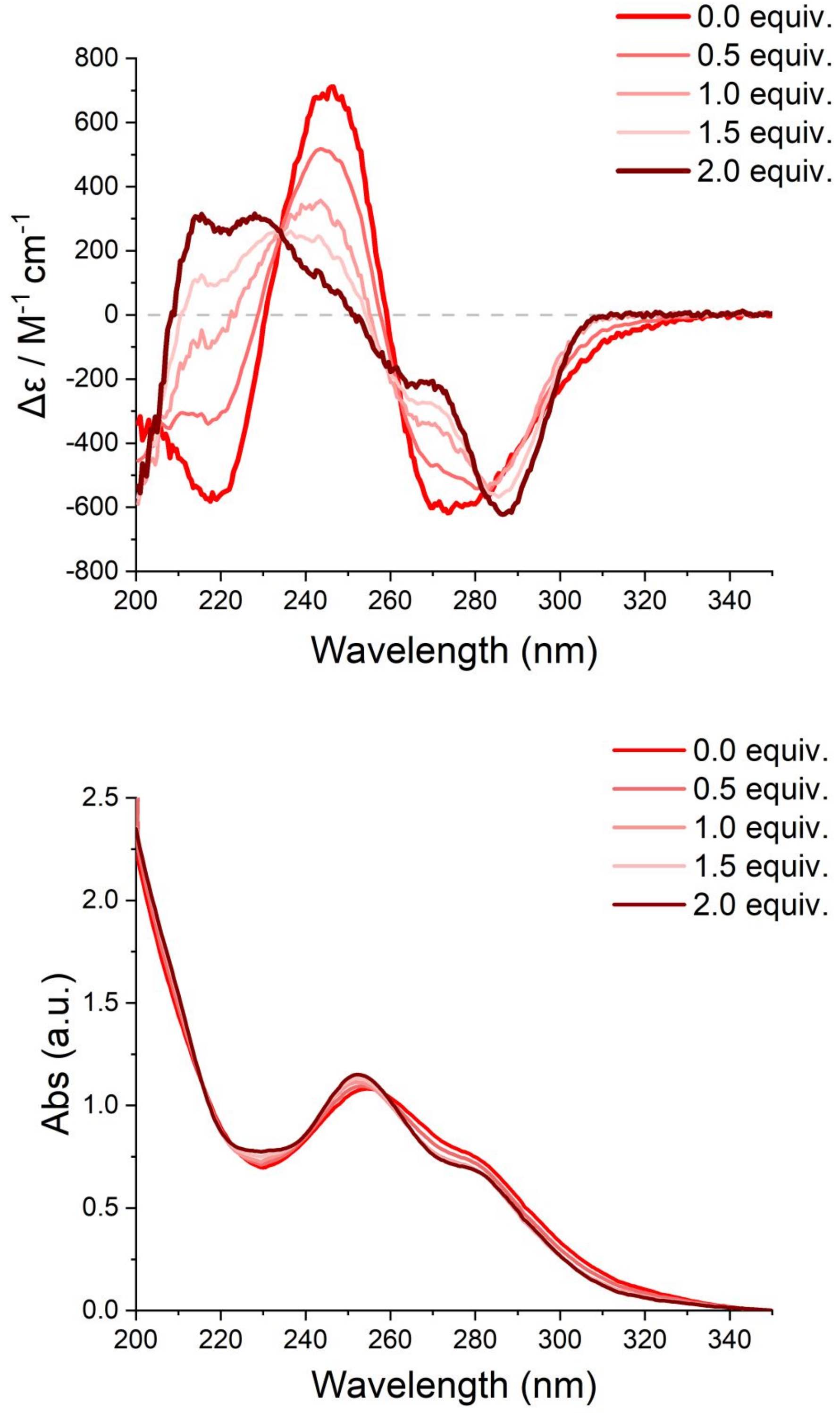


**Figure S24.** CD (top) and UV (bottom) spectra related to the titration of cage ***S*-1** upon incremental addition of $ZnCl_2$ in MeCN/$H_2O$ (1:1).

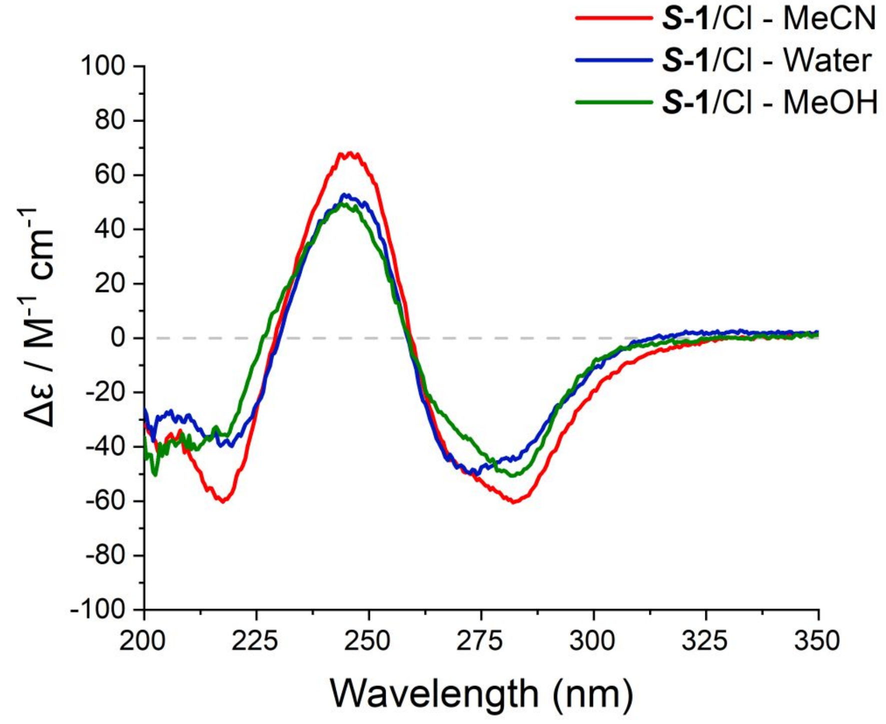


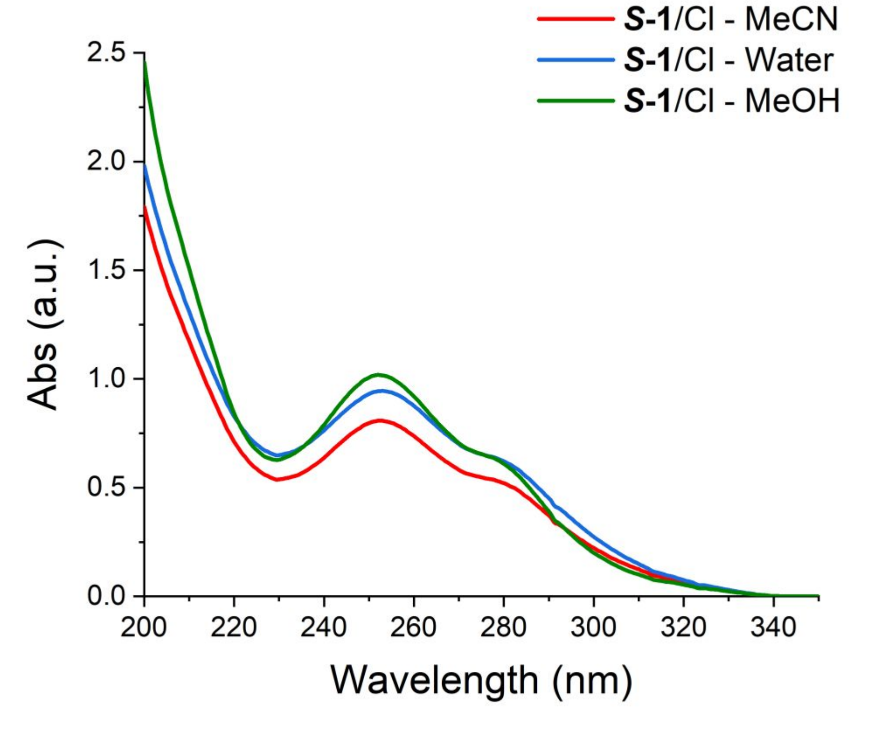


**Figure S21.** CD (top) and UV (bottom) spectra related to the cage ***S*-1** with $Cl^-$ as counterion, in MeCN, MeOH and $H_2O$.

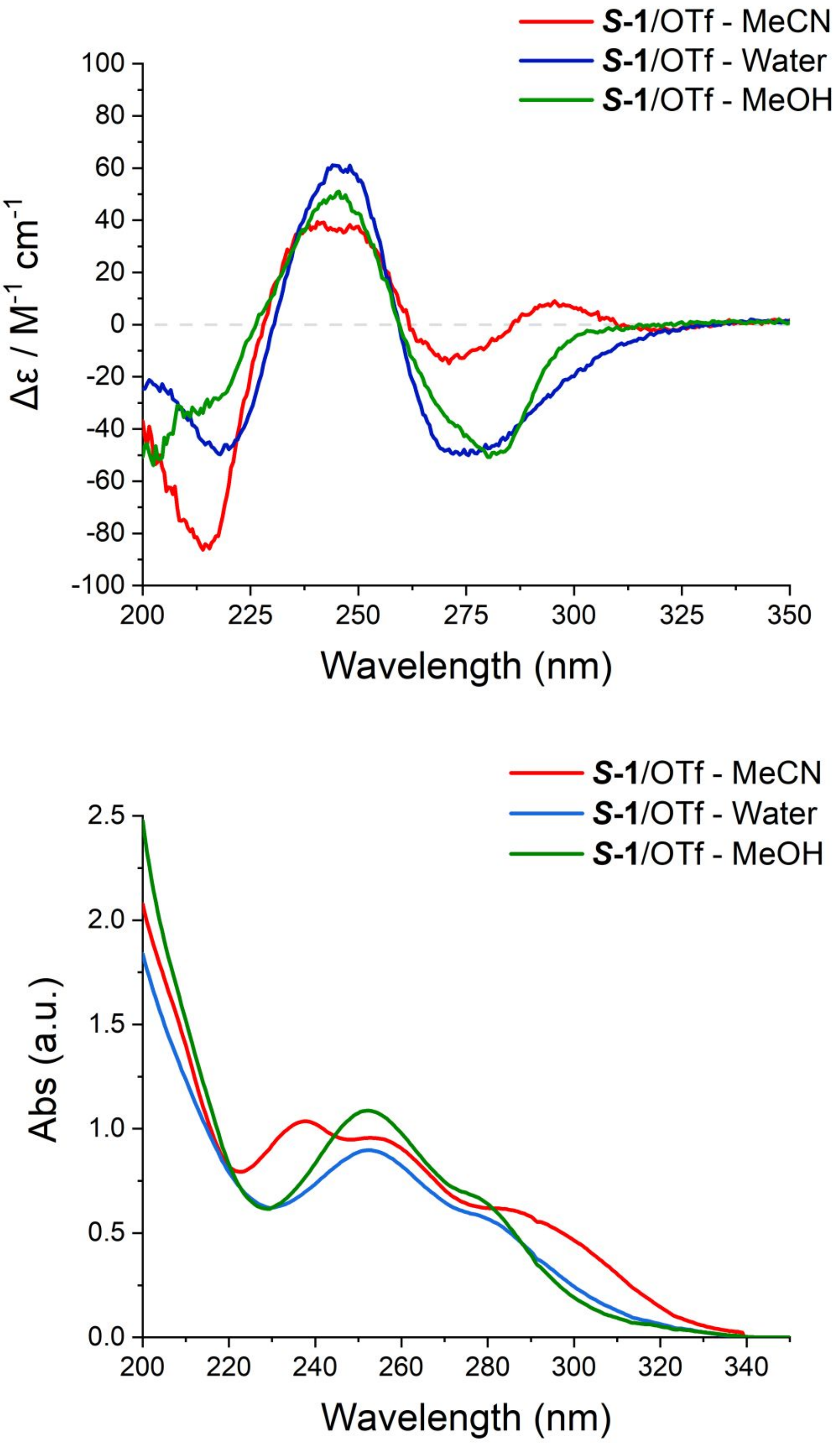


**Figure S26.** CD (top) and UV (bottom) spectra related to the cage ***S*-1** with OTf⁻ as counterion, in MeCN, MeOH and $H_2O$.

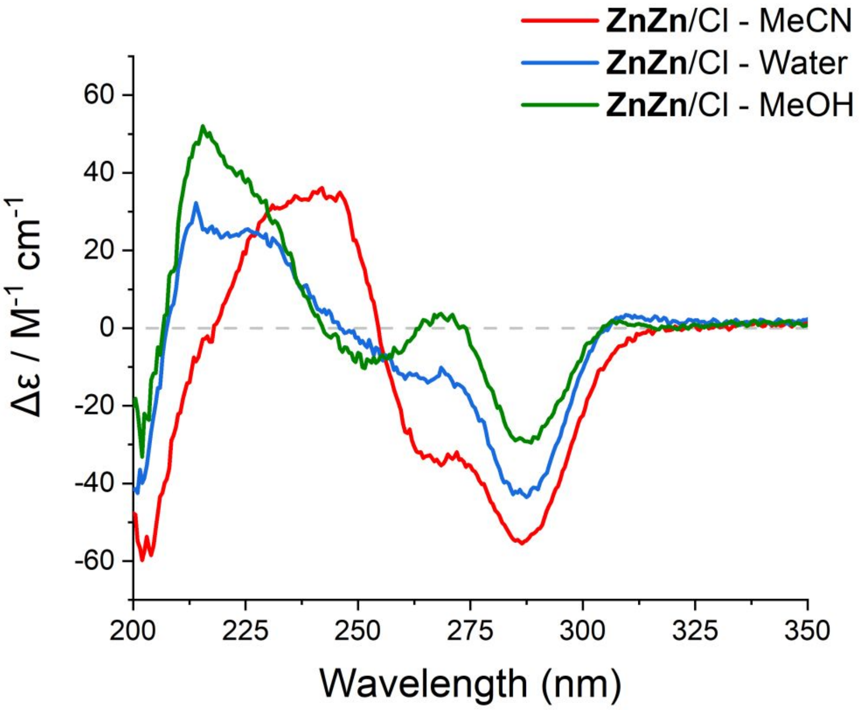


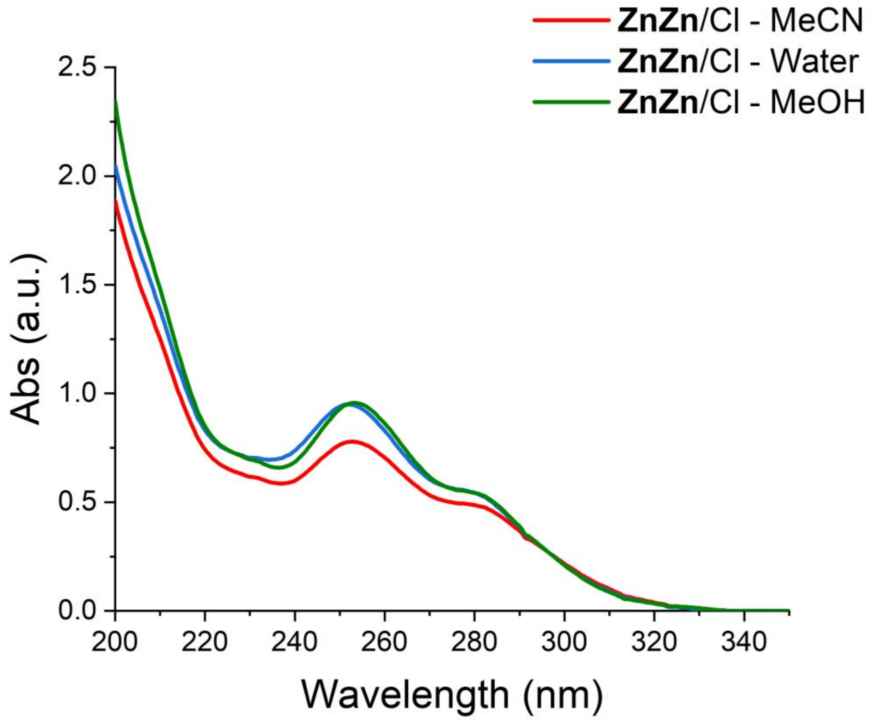


**Figure S27.** CD (top) and UV (bottom) spectra related to the cage **ZnZn** with $Cl^-$ as counterion, in MeCN, MeOH and $H_2O$.

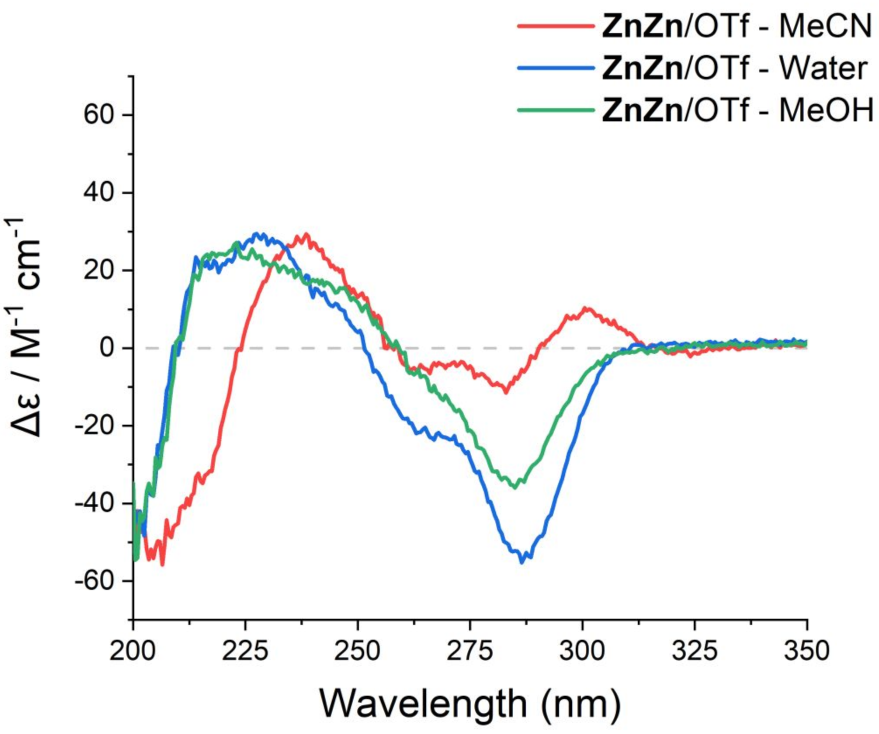


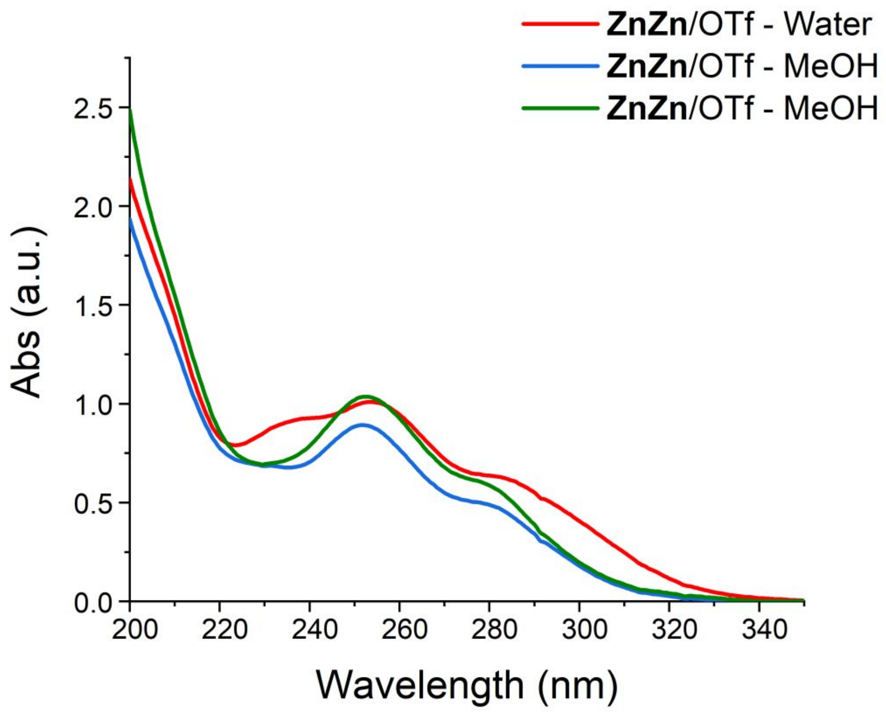


**Figure S28.** CD (top) and UV (bottom) spectra related to the cage **ZnZn** with $OTf^-$ as counterion, in MeCN, MeOH and $H_2O$.

# 4. Computational Section

## 4.1 Conformational analysis

All calculations were performed using the Gaussian 16 package.[S3] Geometry optimizations were carried out at the B3LYP/6-31G(d) level of theory without symmetry constraints, employing default integration grids and loose convergence criteria for the initial conformational screening. Frequency calculations confirmed that all optimized structures correspond to true minima (no imaginary frequencies).

A manual conformational search was conducted for the three species under investigation (***S*-1**, **$ZnH_4$**, and **ZnZn**), starting from two previously reported crystal structures (***S*-1** and **ZnZn**).[S1,S4] For each cage, three levels of nitrogen protonation were examined: unprotonated (A), tertiary-amine protonated (B), and fully protonated (C).

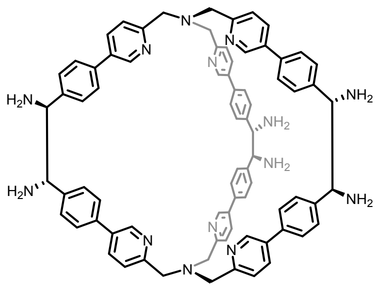


Model A

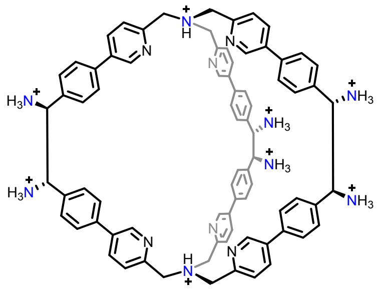


Model B

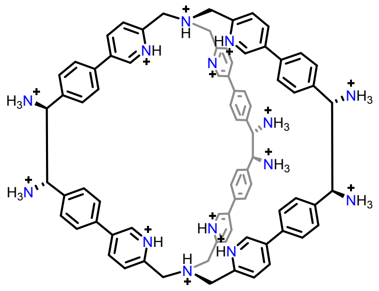


Model C

Twelve starting geometries were generated by systematically varying three stereogenic elements: *i*) the helix helicity of both **TPMA** arms (Λ or Δ), *ii*) the dihedral angle between the pyridine and phenyl rings (+ or -) and *iii*) the relative orientation of the two diamine linkers (*syn* or *anti*). The most stable conformers (***S*-1 B**, **$ZnH_4$ B**, **ZnZn**) were subsequently subjected to time-dependent DFT (TD-DFT) calculations to simulate their electronic spectra. TD-DFT computations were performed using the B3LYP/6-31G(d) basis set, with solvent effects included via the IEF-PCM model (acetonitrile). Excited-state energies and oscillator strengths were obtained for 120 singlet states (TD nstates = 120). Visualization and qualitative analysis of the molecular geometries were carried out using GaussView, Avogadro, and ChimeraX.

a) **TPMA** helix conformation: clockwise *P* (left) and counterclockwise *M* (right).

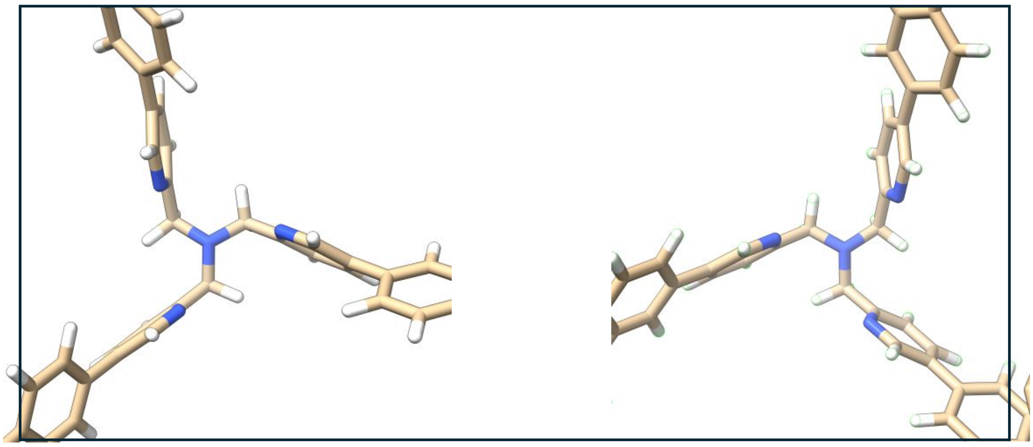

b) Torsion (dihedral) angle defined by four atoms along the pyridine-phenyl bond (atoms 1-2-3-4, see figure below). Positive (*p*) and negative (*m*) values correspond to opposite rotational orientations of the phenyl ring relative to the pyridine unit.

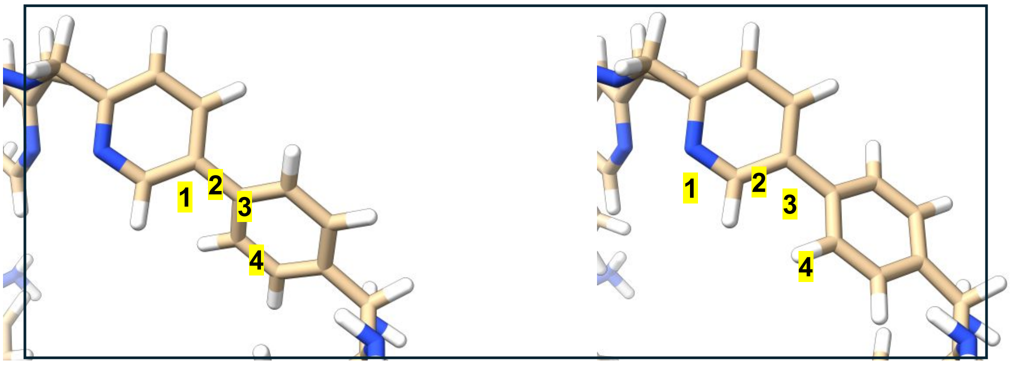


c) Conformation of the two amines: *Syn* (left) and *Anti* (right).

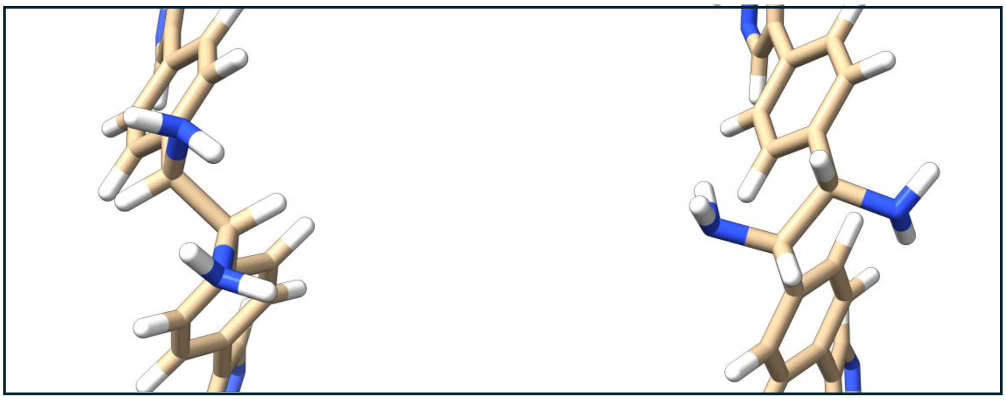

**Table S1.** Classification of conformations assumed by cages stereoisomers (A-L).

| Conformers | Helix | Torsion Angle | Diamine Conformation |
|---|---|---|---|
| **A** | *PP* | *m* | *Anti* |
| **B** | *PP* | *m* | *Syn* |
| **C** | *PP* | *p* | *Anti* |
| **D** | *PP* | *p* | *Syn* |
| **E** | *PM* | *m* | *Anti* |
| **F** | *PM* | *m* | *Syn* |
| **G** | *PM* | *p* | *Anti* |
| **H** | *PM* | *p* | *Syn* |
| **I** | *MM* | *m* | *Anti* |
| **J** | *MM* | *m* | *Syn* |
| **K** | *MM* | *p* | *Anti* |
| **L** | *MM* | *p* | *Syn* |

## 4.2. Energy Calculation of the most stable conformers

**Table S2.** Calculated and relative energies of the examined stereoisomers of cage ***S*-1 A**.

| Conformers | Energy | Relative Energy (kcal/mol) |
|---|---|---|
| **A** | -3782.155963 | 4.9 |
| **B** | *-3782.160841* | *1.9* |
| **C** | -3782.145103 | 11.7 |
| **D** | -3782.141463 | 14.0 |
| **E** | -3782.146111 | 11.1 |
| **F** | **-3782.163812** | **0.0** |
| **G** | -3782.104322 | 37.3 |
| **H** | *-3782.162055* | *1.1* |
| **I** | -3782.108375 | 34.8 |
| **J** | -3782.156807 | 4.4 |
| **K** | -3782.14597 | 11.2 |
| **L** | -3782.135243 | 17.9 |

**Table S3.** Calculated and relative energies of the examined stereoisomers of cage ***S*-1 B**.

| Conformers | Energy | Relative Energy (kcal/mol) |
|---|---|---|
| **A** | *-3783.844905* | *2.6* |
| **B** | -3783.837221 | 7.5 |
| **C** | *-3783.844902* | *2.6* |
| **D** | -3782.301477 | 971.1 |
| **E** | -3783.838699 | 6.5 |
| **F** | *-3783.8449* | *2.6* |
| **G** | -3783.797802 | 32.2 |
| **H** | -3783.842274 | 4.3 |
| **I** | -3783.797287 | 32.5 |
| **J** | **-3783.84911** | **0.0** |
| **K** | -3783.837801 | 7.1 |
| **L** | -3783.837618 | 7.2 |

**Table S4.** Calculated and relative energies of the examined stereoisomers of cage ***S*-1 C**.

| Conformers | Energy | Relative Energy (kcal/mol) |
|---|---|---|
| **A** | -3782.502755 | 4.1 |
| **B** | *-3782.509326* | *0.01* |
| **C** | **-3782.509345** | **0.0** |
| **D** | -3782.507423 | *1.2* |
| **E** | -3782.507712 | *1.0* |
| **F** | *-3782.508982* | *0.2* |
| **G** | -3782.507706 | *1.0* |
| **H** | -3782.507705 | *1.0* |
| **I** | -3782.507843 | *0.9* |
| **J** | -3782.470906 | 24.1 |
| **K** | -3782.470904 | 24.1 |
| **L** | -3782.470898 | 24.1 |

**Table S5.** Calculated and relative energies of the examined stereoisomers of cage **$ZnH_4$ A**.

| Conformers | Energy | Relative Energy (kcal/mol) |
|---|---|---|
| **A** | -5561.188318 | *2.4* |
| **B** | -5561.170444 | 13.6 |
| **C** | -5561.188328 | 2.39 |
| **D** | -5561.188332 | 2.38 |
| **E** | -5561.173774 | 11.52 |
| **F** | -5561.188318 | 2.4 |
| **G** | -5561.095072 | 60.9 |
| **H** | -5561.190896 | *0.8* |
| **I** | -5561.191224 | *0.6* |
| **J** | **-5561.192139** | **0.0** |
| **K** | -5561.175119 | 10.7 |
| **L** | -5561.175118 | 10.7 |

**Table S6.** Calculated and relative energies of the examined stereoisomers of cage **$ZnH_4$ B**.

| Conformers | Energy | Relative Energy (kcal/mol) |
|---|---|---|
| **A** | -5561.803076 | 3.5 |
| **B** | -5561.798547 | 6.4 |
| **C** | -5561.803075 | 3.5 |
| **D** | -5561.800187 | 5.3 |
| **E** | *-5561.805939* | *1.7* |
| **F** | -5561.803073 | 3.5 |
| **G** | -5561.753549 | 34.6 |
| **H** | *-5561.80421* | *2.8* |
| **I** | -5561.753008 | 34.9 |
| **J** | **-5561.808662** | **0.0** |
| **K** | -5561.79334 | 9.6 |
| **L** | -5561.797188 | 7.2 |

**Table S7.** Calculated and relative energies of the examined stereoisomers of cage **ZnZn A**.

| Conformers | Energy | Relative Energy (kcal/mol) |
|---|---|---|
| **A** | -7339.716716 | 3.9 |
| **B** | -7339.715882 | 4.4 |
| **C** | -7339.716722 | 3.9 |
| **D** | -7339.713865 | 5.7 |
| **E** | -7339.718604 | *2.7* |
| **F** | -7339.716725 | 3.9 |
| **G** | -7339.664767 | 36.5 |
| **H** | -7339.719288 | *2.3* |
| **I** | -7339.663135 | 37. |
| **J** | **-7339.722876** | **0.0** |
| **K** | -7339.722852 | *0.02* |
| **L** | -7339.722875 | *0.0006* |

## 4.3. B3LYP/6-31G(d) Minimized Structures

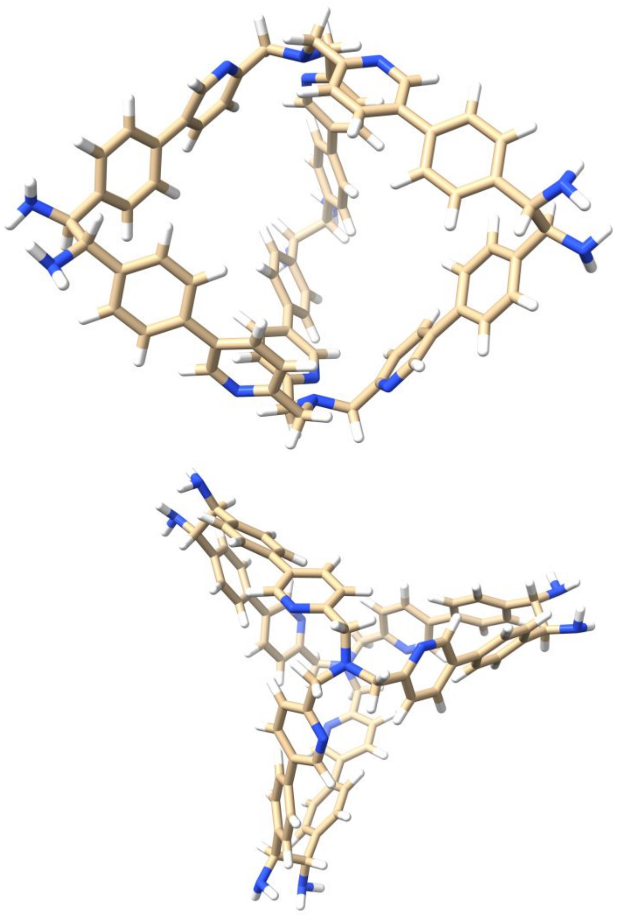

**Figure S29.** B3LYP/6-31G(d) minimized structure for the cage ***S*-1 A**.

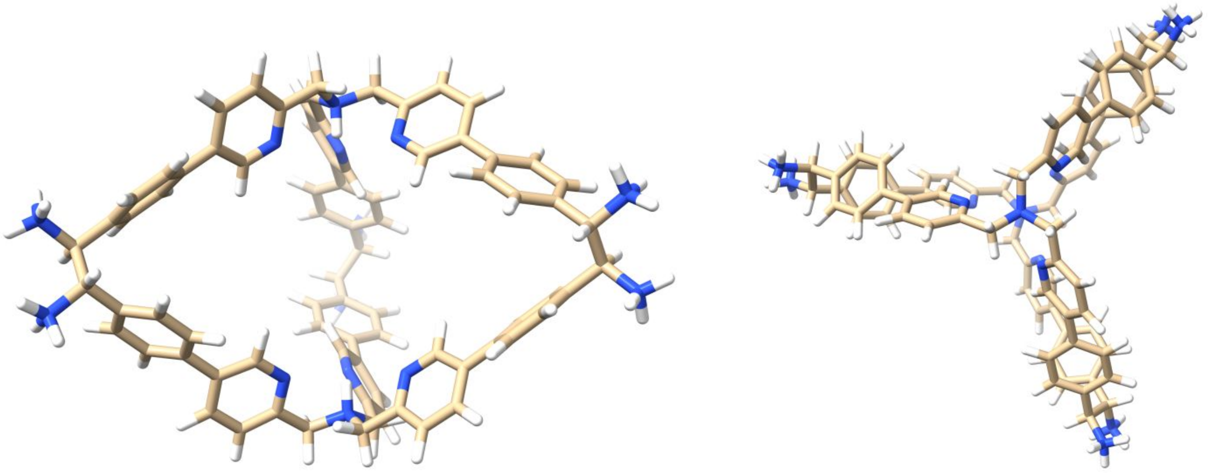

**Figure S30.** B3LYP/6-31G(d) minimized structure for the cage ***S*-1 B**.

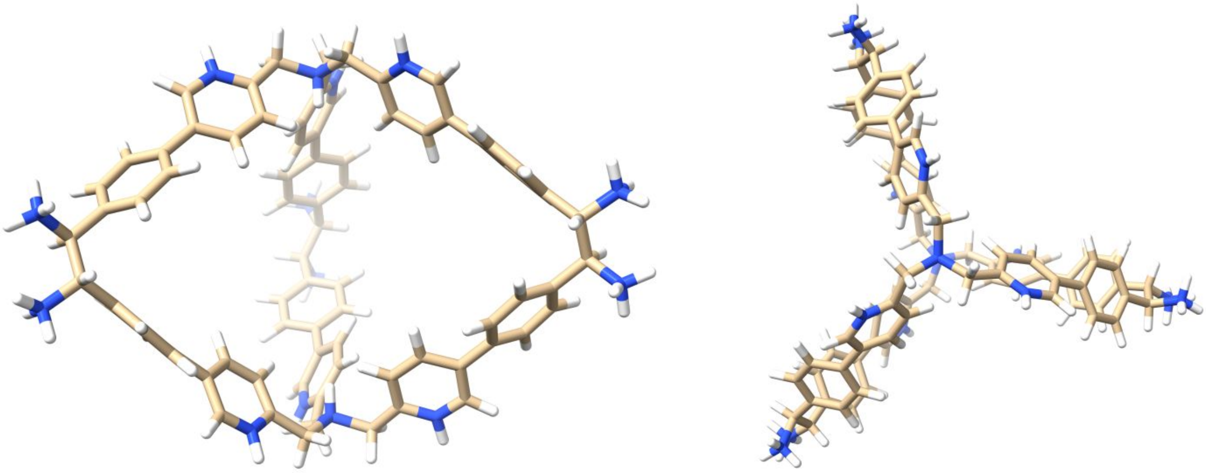

**Figure S31.** B3LYP/6-31G(d) minimized structure for the cage ***S*-1 C**.

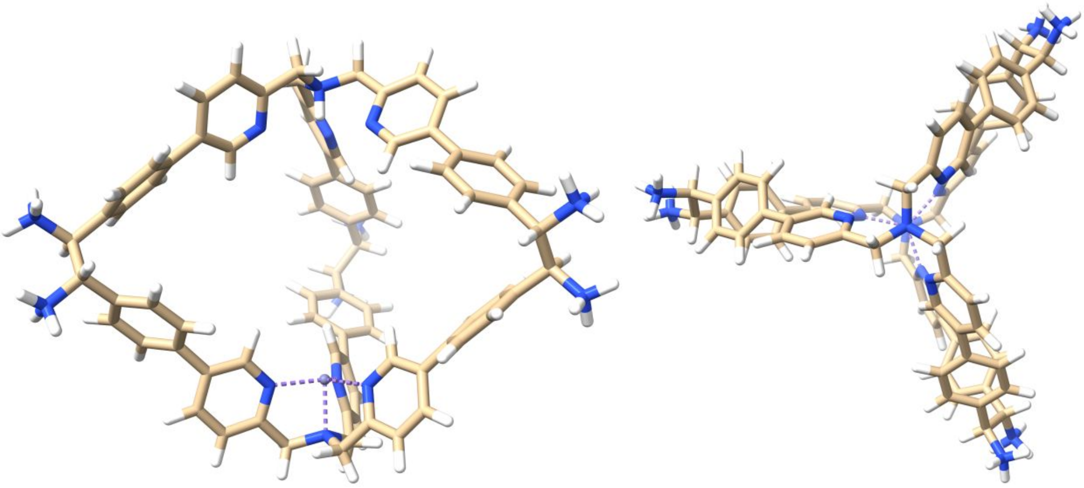

**Figure S32.** B3LYP/6-31G(d) minimized structure for the cage **ZnH$_4$ B**.

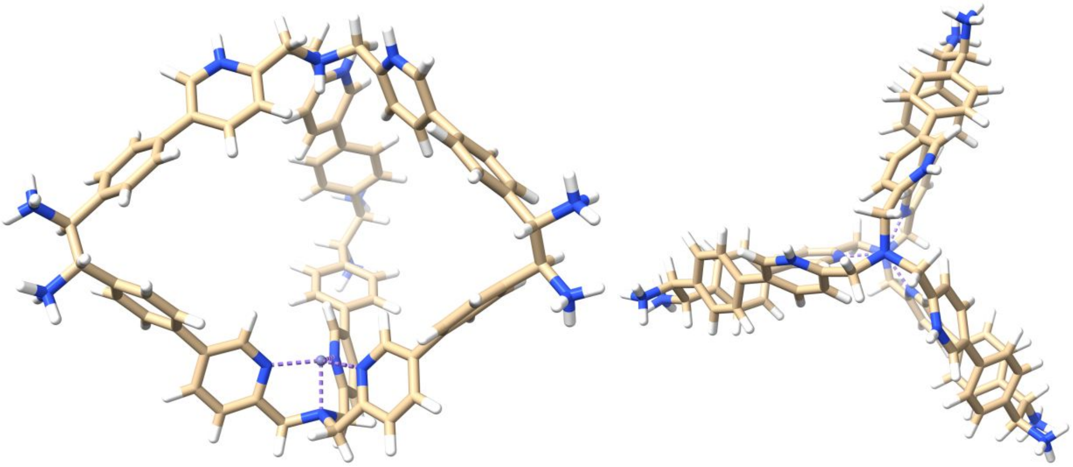

**Figure S33.** B3LYP/6-31G(d) minimized structure for the cage **ZnH$_4$ C**.

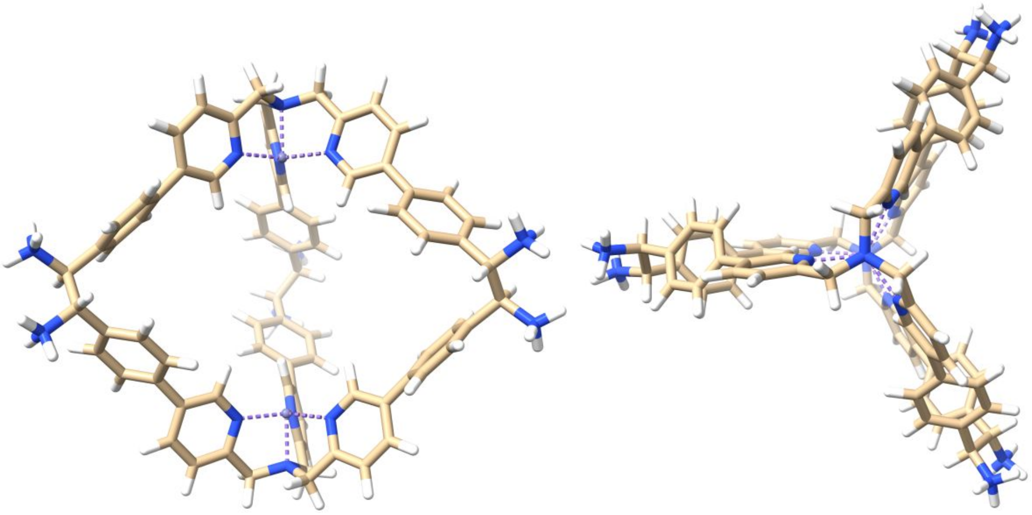

**Figure S34.** B3LYP/6-31G(d) minimized structure for the cage **ZnZn B**.

## 4.4. Structural Parameters of the Lowest-Energy Conformations

The optimized geometries of the lowest-energy conformers of ***S*-1**, **$ZnH_4$**, and **ZnZn** were analyzed to quantify the structural evolution upon sequential Zn(II) coordination. The key metrics include the inter-**TPMA** ($N_{TPMA}$–$N_{TPMA}$) centroid distance, the inter-ring dihedral angles between **TPMA** arms, and the Zn–N coordination distances.

**Table S8.** Inter-**TPMA** distance.

| Structure | $N_{TPMA}$-$N_{TPMA}$ distance (Å) |
|---|---|
| ***S*-1** | 10.947 |
| **$ZnH_4$** | 12.729 |
| **ZnZn** | 13.983 |

**Table S9.** Inter-ring dihedral angles.

| Structure | TPMA 1 | TPMA 2 |
|---|---|---|
| ***S*-1** | 44.04° | 44.07° |
| **$ZnH_4$** | 44.59° | 55.29° |
| **ZnZn** | 56.79° | 56.82° |

| ***S*-1** | | | |
|---|---|---|---|
| **TPMA 1** | | **TPMA 2** | |
| **Arm 1** | 44.07889° | **Arm 1** | 44.05055° |
| **Arm 2** | 43.96592° | **Arm 2** | 44.11232° |
| **Arm 3** | 44.07518° | **Arm 3** | 44.04281° |
| **$ZnH_4$** | | | |
| **TPMA 1** | | **TPMA 2** | |
| **Arm 1** | 44.56583° | **Arm 1** | 55.31788° |
| **Arm 2** | 44.61333° | **Arm 2** | 55.26651° |

| | | | |
|---|---|---|---|
| **Arm 3** | 44.59088° | **Arm 3** | 55.29482° |
| **ZnZn** | | | |
| **TPMA 1** | | **TPMA 2** | |
| **Arm 1** | 56.81340° | **Arm 1** | 56.79630° |
| **Arm 2** | 56.78604° | **Arm 2** | 56.82665° |
| **Arm 3** | 56.77995° | **Arm 3** | 56.83843° |

**Table S1.** Zn–N bond lengths.

| Structure | Zn–N(tertiary) (Å) | Zn–N(pyridyl) (Å) |
|---|---|---|
| ***S*-1** | – | – |
| **$ZnH_4$** | 2.02489 | 2.07580, 2.07581, 2.07566 |
| **ZnZn** (free TPMA) | 2.02272 | 2.05678, 2.05682, 2.05690 |
| **ZnZn** (TPMA metalated) | 2.02272 | 2.05679, 2.05687, 2.05685 |

# 5. TD Calculations

Time-dependent density functional theory (TD-DFT) calculations were performed on the lowest-energy conformers of ***S*-1**, **$ZnH_4$**, and **ZnZn** to interpret the observed CD features in the 200–350 nm region (PCM-MeCN, 120 singlets).[S5] Simulated ECD spectra were obtained by Gaussian convolution of the calculated rotatory strengths (HWHH = 0.11-0.33 eV).

To ensure consistent comparison, an initial series of spectra was generated using a uniform bandwidth of 0.333 eV for all three species, allowing direct visualization of the global spectral evolution across the metalation series. Subsequently, individual broadening factors were applied to each system (0.333 eV for ***S*-1**, 0.11 eV for **$ZnH_4$**, and 0.15 eV for **ZnZn**) to better reproduce the experimental bandwidths and account for the increasing rigidity upon Zn(II) coordination. All theoretical traces were uniformly scaled per species for comparison with the experimental CD data.

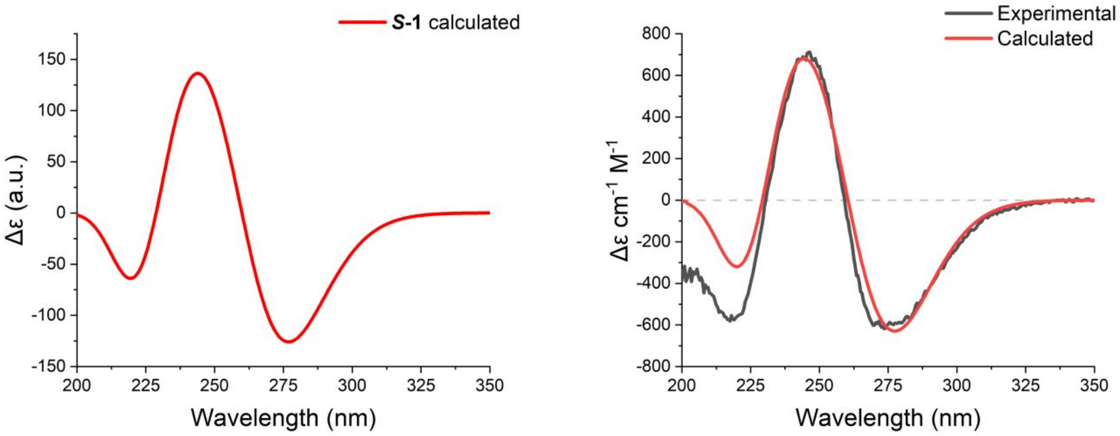


**Figure S35.** Calculated CD spectra (left, HWHH = 0.333 eV) and overlap of experimental molar ellipticity and calculated CD spectra of cage ***S*-1** cage (right, HWHH = 0.333 eV, scale ratio 1:9).

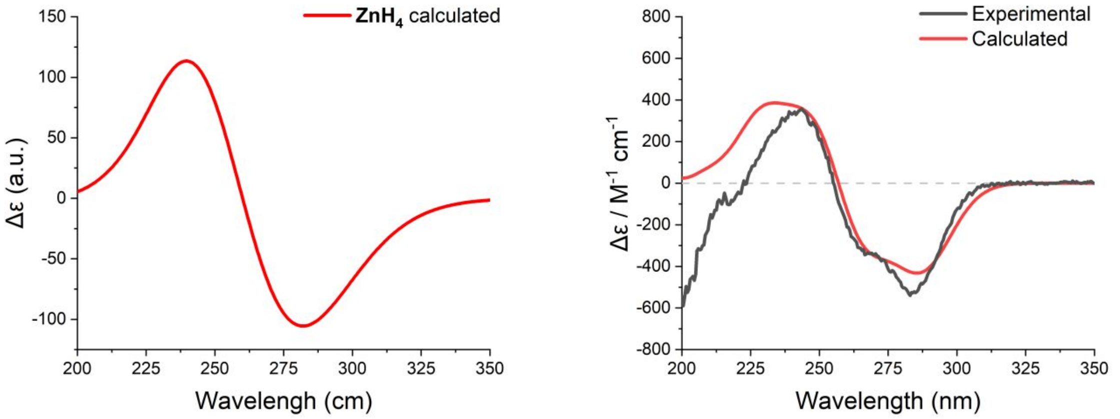


**Figure S36.** Calculated CD spectra (left, HWHH = 0.333 eV) and overlap of experimental molar ellipticity and calculated CD spectra of cage **$ZnH_4$** cage (right, HWHH = 0.11 eV, scale ratio 1:9).

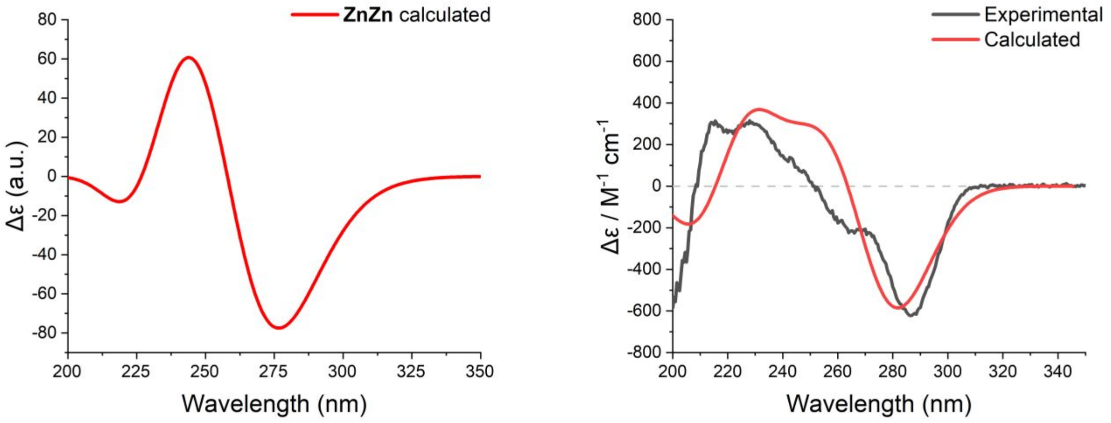


**Figure S37.** Calculated CD spectra (left, HWHH = 0.333 eV) and overlap of experimental molar ellipticity and calculated CD spectra of cage **ZnZn** cage (right, HWHH = 0.15 eV, scale ratio 1:6).

# 6. DFT minimized geometry coordinates

## 6.1 Cage *S*-1 B

| | | | |
|---|---|---|---|
| N | -0.00018900 | 0.00212200 | 5.47328300 |
| N | -2.39722400 | -0.29842800 | 4.06506800 |
| C | -1.00128100 | -1.03676000 | 5.93379900 |
| H | -0.54605400 | -2.00832300 | 5.72687300 |
| H | -1.10330200 | -0.93181300 | 7.01765200 |
| C | -2.34953400 | -0.94591100 | 5.23587500 |
| C | -3.46239700 | -1.58379800 | 5.79470100 |
| H | -3.40026800 | -2.08214100 | 6.75784800 |
| C | -4.66275700 | -1.56668300 | 5.09025500 |
| H | -5.53989800 | -2.04627700 | 5.51516400 |
| C | -4.73828300 | -0.89660100 | 3.85752800 |
| C | -3.56404600 | -0.26952200 | 3.40734000 |
| C | -5.99320900 | -0.86187800 | 3.05488700 |
| C | -6.77145000 | -2.02328800 | 2.88418200 |
| H | -6.45993700 | -2.95652500 | 3.34328700 |
| C | -7.92817600 | -2.00881100 | 2.10702900 |
| H | -8.47653000 | -2.94333500 | 1.99309900 |
| C | -8.35439600 | -0.82093300 | 1.48714000 |
| C | -7.60042800 | 0.34677100 | 1.67328900 |
| H | -7.91778800 | 1.28485100 | 1.22505200 |
| C | -6.43592400 | 0.32228300 | 2.43994300 |
| H | -5.88526000 | 1.24617800 | 2.58654200 |
| C | -9.60903500 | -0.77077900 | 0.62949400 |
| C | -9.49093000 | -1.69451600 | -0.62180800 |
| N | 0.93873600 | 2.22622500 | 4.06394800 |
| C | -0.39955500 | 1.38882000 | 5.93277800 |
| H | -1.46845000 | 1.48034500 | 5.72531700 |
| H | -0.25807100 | 1.42541400 | 7.01667500 |
| C | 0.35385400 | 2.51016700 | 5.23421600 |
| C | 0.35840400 | 3.79341400 | 5.79186300 |
| C | 0.97434300 | 4.82334500 | 5.08668000 |
| H | 0.99804200 | 5.82316700 | 5.51064500 |
| C | 1.59272100 | 4.55213200 | 3.85446000 |
| C | 1.54805700 | 3.22127200 | 3.40551200 |
| C | 2.25123200 | 5.62037100 | 3.05097500 |
| C | 1.63514000 | 6.87507000 | 2.87836300 |
| H | 0.67107500 | 7.07289200 | 3.33683400 |
| C | 2.22650100 | 7.86817100 | 2.09973200 |

| | | | |
|---|---|---|---|
| H | 1.69148500 | 8.81014900 | 1.98392800 |
| C | 3.46821200 | 7.64178700 | 1.48014800 |
| C | 4.10202300 | 6.40505800 | 1.66837900 |
| H | 5.07302300 | 6.20970200 | 1.22043600 |
| C | 3.49811800 | 5.41026900 | 2.43656900 |
| H | 4.02244000 | 4.47138100 | 2.58454500 |
| C | 4.13879600 | 8.70129000 | 0.61989700 |
| C | 3.27817900 | 9.05906000 | -0.63070200 |
| N | 1.45887000 | -1.92308400 | 4.06556000 |
| C | 1.40004600 | -0.34496000 | 5.93412100 |
| H | 2.01353900 | 0.53529800 | 5.72748900 |
| H | 1.35991000 | -0.48598900 | 7.01792900 |
| C | 1.99600300 | -1.55761300 | 5.23611000 |
| C | 3.10549900 | -2.20165800 | 5.79458600 |
| H | 3.50620100 | -1.89834800 | 6.75758100 |
| C | 3.69142300 | -3.24927800 | 5.08988600 |
| H | 4.54599500 | -3.76833800 | 5.51436000 |
| C | 3.14869500 | -3.65010300 | 3.85736000 |
| C | 2.01774100 | -2.94766400 | 3.40761900 |
| C | 3.74712100 | -4.75345000 | 3.05434800 |
| C | 5.14206300 | -4.84413700 | 2.88232900 |
| H | 5.79352500 | -4.10641700 | 3.34069100 |
| C | 5.70907300 | -5.85196500 | 2.10457200 |
| H | 6.79247100 | -5.85731000 | 1.98941000 |
| C | 4.89467400 | -6.81641400 | 1.48529600 |
| C | 3.50646700 | -6.75000200 | 1.67284600 |
| H | 2.85371200 | -7.49498500 | 1.22498000 |
| C | 2.94417900 | -5.73044500 | 2.44020100 |
| H | 1.86883400 | -5.71742500 | 2.58767000 |
| C | 5.47983800 | -7.92640800 | 0.62642600 |
| C | 6.21861700 | -7.35971500 | -0.62494100 |
| N | -0.00553000 | -0.00439100 | -5.47324200 |
| N | -2.39925400 | -0.32225100 | -4.06474500 |
| C | -1.23743700 | 0.74651500 | -5.93370000 |
| H | -1.04341600 | 1.80177900 | -5.72694000 |
| H | -1.30986700 | 0.61887100 | -7.01750100 |
| C | -2.51818100 | 0.31670400 | -5.23515400 |
| C | -3.75682600 | 0.65167200 | -5.79302900 |
| H | -3.82379300 | 1.14981200 | -6.75598400 |
| C | -4.91310300 | 0.33041500 | -5.08785000 |
| H | -5.88350800 | 0.57202200 | -5.51196700 |
| C | -4.81530300 | -0.33744700 | -3.85548000 |
| C | -3.52012400 | -0.64647100 | -3.40639200 |
| C | -6.01970000 | -0.68958600 | -3.05190900 |
| C | -7.06634800 | 0.23689000 | -2.87909000 |
| H | -7.00172300 | 1.21897100 | -3.33742500 |
| C | -8.18058000 | -0.07037400 | -2.10046400 |
| H | -8.94728400 | 0.69496200 | -1.98463400 |
| C | -8.29130400 | -1.32785700 | -1.48125300 |
| C | -7.26667200 | -2.26663400 | -1.66967200 |
| H | -7.33558500 | -3.25476600 | -1.22192700 |
| C | -6.14739600 | -1.94768700 | -2.43771400 |
| H | -5.38093800 | -2.70198900 | -2.58581800 |
| N | 1.46847700 | -1.91603600 | -4.06437600 |
| C | -0.04025300 | -1.44717500 | -5.93210300 |
| H | -1.05074600 | -1.80694000 | -5.72350500 |
| H | 0.10493700 | -1.44718700 | -7.01612300 |
| C | 0.97392400 | -2.33996900 | -5.23396500 |
| C | 1.30430300 | -3.58005300 | -5.79143400 |
| H | 0.90565600 | -3.88834100 | -6.75368400 |
| C | 2.16319500 | -4.41883300 | -5.08696900 |
| H | 2.44038800 | -5.37975200 | -5.51091500 |
| C | 2.69350500 | -3.99858400 | -3.85550300 |
| C | 2.31166800 | -2.72293900 | -3.40651200 |
| C | 3.60368600 | -4.86326600 | -3.05285600 |
| C | 3.32923200 | -6.23392200 | -2.88076000 |
| H | 2.44764000 | -6.67160500 | -3.33894700 |
| C | 4.15561500 | -7.04301800 | -2.10315300 |
| H | 3.87964800 | -8.09063700 | -1.98776300 |
| C | 5.29847500 | -6.50672700 | -1.48404700 |
| C | 5.59455700 | -5.14887400 | -1.67167700 |
| H | 6.48343300 | -4.71176800 | -1.22398200 |
| C | 4.75572400 | -4.34141700 | -2.43896000 |
| H | 5.02226400 | -3.29954400 | -2.58662900 |
| N | 0.91861700 | 2.22704400 | -4.06505900 |
| C | 1.26097000 | 0.68627100 | -5.93426800 |

| | | | |
|---|---|---|---|
| H | 2.07753400 | -0.00989100 | -5.72783700 |
| H | 1.18621200 | 0.81265700 | -7.01805500 |
| C | 1.53017000 | 2.01029600 | -5.23604800 |
| C | 2.43937200 | 2.91512100 | -5.79485900 |
| C | 2.74004500 | 4.07730400 | -5.09031500 |
| H | 3.43408800 | 4.79673800 | -5.51532000 |
| C | 2.11381300 | 4.32702700 | -3.85751200 |
| C | 1.19903300 | 3.36002300 | -3.40723100 |
| C | 2.41148300 | 5.54682800 | -3.05514800 |
| C | 3.73719100 | 5.99059500 | -2.88416800 |
| H | 4.55507500 | 5.44333100 | -3.34272600 |
| C | 4.02864400 | 7.11037300 | -2.10743600 |
| H | 5.07483100 | 7.39217800 | -1.99312300 |
| C | 2.99536500 | 7.83549600 | -1.48812300 |
| C | 1.66998100 | 7.41685400 | -1.67463400 |
| H | 0.84893300 | 7.97112100 | -1.22706900 |
| C | 1.38613700 | 6.28695800 | -2.44091700 |
| H | 0.34960300 | 6.00003900 | -2.58785300 |
| H | 5.12640600 | 8.35152300 | 0.30682100 |
| H | 2.33536400 | 9.51597000 | -0.31761400 |
| H | -9.80124900 | 0.25938900 | 0.31711500 |
| H | -9.41518700 | -2.73973000 | -0.30949000 |
| H | 7.08406100 | -6.76883100 | -0.31253900 |
| H | 4.68490600 | -8.60933600 | 0.31425000 |
| N | 3.92426000 | 10.13979400 | -1.55732300 |
| H | 3.32099100 | 10.24866800 | -2.38817800 |
| H | 4.01999300 | 11.08401400 | -1.15514200 |
| N | 4.45347400 | 9.92265100 | 1.54361000 |
| H | 4.95573200 | 9.57211100 | 2.37486900 |
| H | 5.05711500 | 10.65384100 | 1.13935000 |
| N | -10.82193300 | -1.10979000 | 1.55581800 |
| H | -10.76769500 | -0.50004500 | 2.38729400 |
| H | -11.75795800 | -0.95250800 | 1.15382300 |
| N | -10.75155400 | -1.67427100 | -1.54617600 |
| H | -10.54564400 | -2.25014700 | -2.37810700 |
| H | -11.61635500 | -2.06409800 | -1.14290700 |
| N | 6.83488100 | -8.45950500 | -1.54948600 |
| H | 7.22960500 | -7.99183000 | -2.38111000 |
| H | 7.60628300 | -9.01145800 | -1.14602000 |
| N | 6.38260200 | -8.80604600 | 1.55119600 |
| H | 5.82880800 | -9.06572700 | 2.38306100 |
| H | 6.71588200 | -9.69430700 | 1.14819600 |
| H | -0.10450800 | 3.98996000 | 6.75463900 |
| H | 0.68421400 | 3.49249300 | -2.45779600 |
| H | -3.55589100 | 0.26216500 | 2.45798000 |
| H | -3.37675100 | -1.15909900 | -2.45735500 |
| H | 2.00456300 | 2.94722800 | 2.45658100 |
| H | 2.90337500 | 2.72368700 | -6.75816300 |
| H | 1.55316200 | -3.20671000 | 2.45840100 |
| H | 2.68439700 | -2.34133700 | -2.45810900 |
| H | 0.00006100 | 0.00170400 | 4.43875800 |
| H | -0.00526400 | -0.00369900 | -4.43868300 |
| H | 4.84507800 | 9.85371200 | -1.92008400 |
| H | 3.60278000 | 10.37691300 | 1.90599100 |
| H | -10.78895200 | -2.07382900 | 1.91758700 |
| H | -10.96498800 | -0.73337600 | -1.90742100 |
| H | 6.12878100 | -9.11674200 | -1.91116400 |
| H | 7.20030100 | -8.29402300 | 1.91247300 |

## 6.2 Cage $ZnH_4$ B

| | | | |
|---|---|---|---|
| N | -6.99140600 | 0.00032700 | -0.00182600 |
| N | -5.07114300 | -0.55960000 | 1.97529700 |
| C | -7.38204100 | 0.17578300 | 1.42745900 |
| H | -7.46001000 | 1.25193200 | 1.62452800 |
| H | -8.37468300 | -0.24630600 | 1.62271600 |
| C | -6.35671900 | -0.41196300 | 2.39389100 |

| | | | |
|---|---|---|---|
| C | -6.72327100 | -0.72667100 | 3.70262900 |
| C | -5.76018800 | -1.18269300 | 4.59806200 |
| C | -4.42704300 | -1.35516600 | 4.17837900 |
| C | -4.14334600 | -1.03233800 | 2.84647400 |
| C | -3.38780500 | -1.87066000 | 5.13340000 |
| C | -3.18831900 | -1.23719800 | 6.37506900 |
| H | -3.74812700 | -0.34141600 | 6.62828400 |
| C | -2.27468100 | -1.74093900 | 7.30409900 |
| H | -2.16404900 | -1.20225100 | 8.24414300 |
| C | -1.54638000 | -2.91290000 | 7.03025500 |
| C | -1.74190400 | -3.54798600 | 5.79405700 |
| H | -1.21555700 | -4.46950300 | 5.55899300 |
| C | -2.63980200 | -3.02726700 | 4.85720500 |
| H | -2.79677500 | -3.56884100 | 3.92898400 |
| C | -0.61462400 | -3.54923300 | 8.06593000 |
| C | 0.61014700 | -2.63560800 | 8.40944200 |
| N | -5.07007400 | -1.43171900 | -1.47419800 |
| C | -7.38155800 | -1.32525600 | -0.56470800 |
| H | -7.46014500 | -2.03396100 | 0.26870400 |
| H | -8.37386000 | -1.28339600 | -1.02862000 |
| C | -6.35550900 | -1.86837800 | -1.55616900 |
| C | -6.72136300 | -2.84474900 | -2.48302400 |
| C | -5.75774300 | -3.39212500 | -3.32510000 |
| C | -4.42475200 | -2.94202700 | -3.26413800 |
| C | -4.14175200 | -1.94972400 | -2.31867600 |
| C | -3.38502400 | -3.51131000 | -4.18756300 |
| C | -3.18486200 | -4.90330300 | -4.25902600 |
| H | -3.74440000 | -5.57039400 | -3.60953900 |
| C | -2.27085300 | -5.45604400 | -5.15938900 |
| H | -2.15960900 | -6.53942600 | -5.16223600 |
| C | -1.54283800 | -4.63303300 | -6.03777000 |
| C | -1.73898700 | -3.24496500 | -5.97040800 |
| H | -1.21281800 | -2.58074400 | -6.65117600 |
| C | -2.63724700 | -2.69392000 | -5.05141700 |
| H | -2.79465200 | -1.61933400 | -5.05684500 |
| C | -0.61073600 | -5.21191300 | -7.10633500 |
| C | 0.61421700 | -5.96571700 | -6.48657300 |
| N | -5.07026000 | 1.99195100 | -0.50468300 |
| C | -7.38132500 | 1.15054200 | -0.86855900 |
| H | -7.45920600 | 0.78317900 | -1.89909300 |
| H | -8.37388300 | 1.53110500 | -0.60092100 |
| C | -6.35559800 | 2.28099800 | -0.84246500 |
| C | -6.72158500 | 3.57182800 | -1.22456700 |
| C | -5.75817800 | 4.57500800 | -1.27698300 |
| C | -4.42528300 | 4.29744000 | -0.91710300 |
| C | -4.14214900 | 2.98250500 | -0.53050400 |
| C | -3.38582500 | 5.38205000 | -0.94775600 |
| C | -3.18578300 | 6.14055800 | -2.11713700 |
| H | -3.74522900 | 5.91195600 | -3.01973900 |
| C | -2.27195500 | 7.19682700 | -2.14509800 |
| H | -2.16080300 | 7.74145600 | -3.08164800 |
| C | -1.54403200 | 7.54560600 | -0.99297600 |
| C | -1.74011000 | 6.79266300 | 0.17508900 |
| H | -1.21405000 | 7.04978300 | 1.09085700 |
| C | -2.63817500 | 5.72109500 | 0.19225200 |
| H | -2.79554800 | 5.18806200 | 1.12533800 |
| C | -0.61187100 | 8.76039500 | -0.95968200 |
| C | 0.61292000 | 8.60052500 | -1.92248900 |
| N | 6.99138500 | 0.00006300 | 0.00164100 |
| N | 5.06991600 | -0.87859300 | 1.85803500 |
| C | 7.38130000 | -1.07248100 | 0.96281200 |
| H | 7.45965800 | -2.01256700 | 0.40323800 |
| H | 8.37366500 | -0.88314700 | 1.38824700 |
| C | 6.35526400 | -1.26567900 | 2.07665900 |
| C | 6.72102500 | -1.89017400 | 3.26930900 |
| H | 7.75354900 | -2.18401200 | 3.43732900 |
| C | 5.75739200 | -2.13582000 | 4.24313700 |
| C | 4.42449900 | -1.72920800 | 4.04008100 |
| C | 4.14160700 | -1.09559000 | 2.82468400 |
| C | 3.38478600 | -1.96933000 | 5.09797800 |
| C | 3.18422100 | -3.26359500 | 5.61514400 |
| H | 3.74343800 | -4.10487600 | 5.21583100 |
| C | 2.27015300 | -3.49567000 | 6.64577000 |
| H | 2.15856800 | -4.52006500 | 6.99829200 |
| C | 1.54250600 | -2.43287500 | 7.21137200 |
| C | 1.73912000 | -1.14097500 | 6.69942500 |

| | | | | | | | |
|---|---|---|---|---|---|---|---|
| H | 1.21331200 | -0.29230600 | 7.12927900 | C | 1.74245600 | -5.23156500 | -4.33702900 |
| C | 2.63740200 | -0.91652200 | 5.65167100 | H | 1.21683000 | -6.02834200 | -3.81707800 |
| H | 2.79516100 | 0.10220400 | 5.30983700 | C | 2.64027700 | -4.43604400 | -3.61859800 |
| N | 5.07058900 | 2.04755200 | -0.16624600 | H | 2.79788700 | -4.64925500 | -2.56539300 |
| C | 7.38156900 | 1.36862000 | 0.45001400 | N | 1.48604600 | -6.73407000 | -7.52883900 |
| H | 7.45958500 | 1.35407000 | 1.54396200 | N | -1.48242400 | -6.08586800 | -8.06192500 |
| H | 8.37412900 | 1.64208900 | 0.07362100 | N | -1.48665900 | -3.94026700 | 9.30021900 |
| C | 6.35595900 | 2.43010400 | 0.06003000 | N | 1.48155700 | -3.15411600 | 9.59630000 |
| C | 6.72210700 | 3.77511400 | 0.00452300 | N | 1.48471900 | 9.88734300 | -2.06720900 |
| H | 7.75465400 | 4.06728000 | 0.17529600 | N | -1.48349000 | 10.02510900 | -1.23838200 |
| C | 5.75883000 | 4.74152800 | -0.27006800 | H | -3.13633400 | -1.13966900 | 2.45503700 |
| C | 4.42589900 | 4.36271600 | -0.52097900 | H | -3.13490400 | -1.55675500 | -2.21551100 |
| C | 4.14260600 | 2.99342900 | -0.46194300 | H | 7.75554300 | -1.88326100 | -3.60727300 |
| C | 3.38653100 | 5.39916300 | -0.84237400 | H | -3.13539100 | 2.69689200 | -0.24124200 |
| C | 3.18644300 | 6.49470400 | 0.01934800 | Zn | 4.96866500 | 0.00003000 | 0.00104600 |
| H | 3.74582100 | 6.56986200 | 0.94745100 | Zn | -4.96869000 | 0.00031800 | -0.00127500 |
| C | 2.27272200 | 7.50346000 | -0.29544600 | H | -0.25855700 | -4.51277000 | 7.69125800 |
| H | 2.16157000 | 8.32141900 | 0.41497400 | H | -1.87482600 | -3.13092900 | 9.80817800 |
| C | 1.54491700 | 7.46148400 | -1.49854300 | H | -1.03111100 | -4.55688900 | 9.99139200 |
| C | 1.74106600 | 6.37165300 | -2.36083400 | H | 0.25399900 | -1.66369400 | 8.76172000 |
| H | 1.21516400 | 6.31927300 | -3.31066000 | H | 1.86977000 | -4.09770100 | 9.44578400 |
| C | 2.63905100 | 5.35192100 | -2.03089700 | H | 1.02550000 | -3.14522500 | 10.52225400 |
| H | 2.79648800 | 4.54610500 | -2.74182200 | H | -1.87155400 | 10.06040100 | -2.19331000 |
| N | 5.07090900 | -1.16872200 | -1.68832200 | H | -1.02767800 | 10.93186800 | -1.04997700 |
| C | 7.38197800 | -0.29599700 | -1.40761100 | H | -0.25579700 | 8.91770700 | 0.06208800 |
| H | 7.46030300 | 0.65866600 | -1.94193400 | H | 1.87280600 | 10.22910800 | -1.17484500 |
| H | 8.37447600 | -0.75886200 | -1.45591800 | H | 1.02903400 | 10.68471000 | -2.53839500 |
| C | 6.35645500 | -1.16426400 | -2.13229500 | H | 0.25684600 | 8.41940700 | -2.94031100 |
| C | 6.72287200 | -1.88486900 | -3.26924500 | H | -0.25479500 | -4.40579500 | -7.75367300 |
| C | 5.75970400 | -2.60568600 | -3.96925500 | H | -1.87016400 | -6.93052900 | -7.61479900 |
| C | 4.42661800 | -2.63337800 | -3.51618500 | H | -1.02678600 | -6.37610000 | -8.94147900 |
| C | 4.14304200 | -1.89758600 | -2.35995500 | H | 0.25831300 | -6.75680500 | -5.82093300 |
| C | 3.38738800 | -3.42985900 | -4.25338400 | H | 1.87403700 | -6.13187900 | -8.27082900 |
| C | 3.18710900 | -3.23076400 | -5.63289700 | H | 1.03042400 | -7.54071400 | -7.98402100 |
| H | 3.74622200 | -2.46417800 | -6.16173200 | H | 2.30596600 | -7.13049000 | -7.03981600 |
| C | 2.27353200 | -4.00768600 | -6.34938400 | H | -2.30249600 | -5.52952600 | -8.35607200 |
| H | 2.16215700 | -3.80093300 | -7.41284600 | H | -2.30647500 | -4.47332000 | 8.96508000 |
| C | 1.54609700 | -5.02911600 | -5.71187800 | H | 2.30132200 | -2.53227300 | 9.69556400 |

| | | | |
|---|---|---|---|
| H | 2.30458600 | 9.66187600 | -2.65503800 |
| H | -2.30336800 | 10.00168700 | -0.60925000 |
| H | -6.05364100 | -1.43046200 | 5.61444500 |
| H | -7.75597500 | -0.61594800 | 4.02191100 |
| H | 6.05023800 | -2.61829400 | 5.17157500 |
| H | 3.13485700 | -0.75667900 | 2.60017400 |
| H | -6.05064600 | -4.14870300 | -4.04782700 |
| H | -7.75395100 | -3.17689900 | -2.54717900 |
| H | 3.13608600 | -1.87275300 | -1.95469700 |
| H | 6.05305500 | -3.16849400 | -4.85114600 |
| H | -6.05120700 | 5.57918200 | -1.57075400 |
| H | -7.75411100 | 3.79326400 | -1.48056100 |
| H | 3.13577800 | 2.62982500 | -0.64333900 |
| H | 6.05197600 | 5.78673400 | -0.31645700 |

## 6.3 Cage ZnZn B

| | | | |
|---|---|---|---|
| N | -6.99140600 | 0.00032700 | -0.00182600 |
| N | -5.07114300 | -0.55960000 | 1.97529700 |
| C | -7.38204100 | 0.17578300 | 1.42745900 |
| H | -7.46001000 | 1.25193200 | 1.62452800 |
| H | -8.37468300 | -0.24630600 | 1.62271600 |
| C | -6.35671900 | -0.41196300 | 2.39389100 |
| C | -6.72327100 | -0.72667100 | 3.70262900 |
| C | -5.76018800 | -1.18269300 | 4.59806200 |
| C | -4.42704300 | -1.35516600 | 4.17837900 |
| C | -4.14334600 | -1.03233800 | 2.84647400 |
| C | -3.38780500 | -1.87066000 | 5.13340000 |
| C | -3.18831900 | -1.23719800 | 6.37506900 |
| H | -3.74812700 | -0.34141600 | 6.62828400 |
| C | -2.27468100 | -1.74093900 | 7.30409900 |
| H | -2.16404900 | -1.20225100 | 8.24414300 |
| C | -1.54638000 | -2.91290000 | 7.03025500 |
| C | -1.74190400 | -3.54798600 | 5.79405700 |
| H | -1.21555700 | -4.46950300 | 5.55899300 |
| C | -2.63980200 | -3.02726700 | 4.85720500 |
| H | -2.79677500 | -3.56884100 | 3.92898400 |
| C | -0.61462400 | -3.54923300 | 8.06593000 |
| C | 0.61014700 | -2.63560800 | 8.40944200 |
| N | -5.07007400 | -1.43171900 | -1.47419800 |
| C | -7.38155800 | -1.32525600 | -0.56470800 |
| H | -7.46014500 | -2.03396100 | 0.26870400 |
| H | -8.37386000 | -1.28339600 | -1.02862000 |
| C | -6.35550900 | -1.86837800 | -1.55616900 |
| C | -6.72136300 | -2.84474900 | -2.48302400 |
| C | -5.75774300 | -3.39212500 | -3.32510000 |
| C | -4.42475200 | -2.94202700 | -3.26413800 |
| C | -4.14175200 | -1.94972400 | -2.31867600 |
| C | -3.38502400 | -3.51131000 | -4.18756300 |
| C | -3.18486200 | -4.90330300 | -4.25902600 |
| H | -3.74440000 | -5.57039400 | -3.60953900 |
| C | -2.27085300 | -5.45604400 | -5.15938900 |
| H | -2.15960900 | -6.53942600 | -5.16223600 |
| C | -1.54283800 | -4.63303300 | -6.03777000 |
| C | -1.73898700 | -3.24496500 | -5.97040800 |
| H | -1.21281800 | -2.58074400 | -6.65117600 |
| C | -2.63724700 | -2.69392000 | -5.05141700 |
| H | -2.79465200 | -1.61933400 | -5.05684500 |
| C | -0.61073600 | -5.21191300 | -7.10633500 |
| C | 0.61421700 | -5.96571700 | -6.48657300 |
| N | -5.07026000 | 1.99195100 | -0.50468300 |
| C | -7.38132500 | 1.15054200 | -0.86855900 |
| H | -7.45920600 | 0.78317900 | -1.89909300 |

| | | | |
|---|---|---|---|
| H | -8.37388300 | 1.53110500 | -0.60092100 |
| C | -6.35559800 | 2.28099800 | -0.84246500 |
| C | -6.72158500 | 3.57182800 | -1.22456700 |
| C | -5.75817800 | 4.57500800 | -1.27698300 |
| C | -4.42528300 | 4.29744000 | -0.91710300 |
| C | -4.14214900 | 2.98250500 | -0.53050400 |
| C | -3.38582500 | 5.38205000 | -0.94775600 |
| C | -3.18578300 | 6.14055800 | -2.11713700 |
| H | -3.74522900 | 5.91195600 | -3.01973900 |
| C | -2.27195500 | 7.19682700 | -2.14509800 |
| H | -2.16080300 | 7.74145600 | -3.08164800 |
| C | -1.54403200 | 7.54560600 | -0.99297600 |
| C | -1.74011000 | 6.79266300 | 0.17508900 |
| H | -1.21405000 | 7.04978300 | 1.09085700 |
| C | -2.63817500 | 5.72109500 | 0.19225200 |
| H | -2.79554800 | 5.18806200 | 1.12533800 |
| C | -0.61187100 | 8.76039500 | -0.95968200 |
| C | 0.61292000 | 8.60052500 | -1.92248900 |
| N | 6.99138500 | 0.00006300 | 0.00164100 |
| N | 5.06991600 | -0.87859300 | 1.85803500 |
| C | 7.38130000 | -1.07248100 | 0.96281200 |
| H | 7.45965800 | -2.01256700 | 0.40323800 |
| H | 8.37366500 | -0.88314700 | 1.38824700 |
| C | 6.35526400 | -1.26567900 | 2.07665900 |
| C | 6.72102500 | -1.89017400 | 3.26930900 |
| H | 7.75354900 | -2.18401200 | 3.43732900 |
| C | 5.75739200 | -2.13582000 | 4.24313700 |
| C | 4.42449900 | -1.72920800 | 4.04008100 |
| C | 4.14160700 | -1.09559000 | 2.82468400 |
| C | 3.38478600 | -1.96933000 | 5.09797800 |
| C | 3.18422100 | -3.26359500 | 5.61514400 |
| H | 3.74343800 | -4.10487600 | 5.21583100 |
| C | 2.27015300 | -3.49567000 | 6.64577000 |
| H | 2.15856800 | -4.52006500 | 6.99829200 |
| C | 1.54250600 | -2.43287500 | 7.21137200 |
| C | 1.73912000 | -1.14097500 | 6.69942500 |
| H | 1.21331200 | -0.29230600 | 7.12927900 |
| C | 2.63740200 | -0.91652200 | 5.65167100 |

| | | | |
|---|---|---|---|
| H | 2.79516100 | 0.10220400 | 5.30983700 |
| N | 5.07058900 | 2.04755200 | -0.16624600 |
| C | 7.38156900 | 1.36862000 | 0.45001400 |
| H | 7.45958500 | 1.35407000 | 1.54396200 |
| H | 8.37412900 | 1.64208900 | 0.07362100 |
| C | 6.35595900 | 2.43010400 | 0.06003000 |
| C | 6.72210700 | 3.77511400 | 0.00452300 |
| H | 7.75465400 | 4.06728000 | 0.17529600 |
| C | 5.75883000 | 4.74152800 | -0.27006800 |
| C | 4.42589900 | 4.36271600 | -0.52097900 |
| C | 4.14260600 | 2.99342900 | -0.46194300 |
| C | 3.38653100 | 5.39916300 | -0.84237400 |
| C | 3.18644300 | 6.49470400 | 0.01934800 |
| H | 3.74582100 | 6.56986200 | 0.94745100 |
| C | 2.27272200 | 7.50346000 | -0.29544600 |
| H | 2.16157000 | 8.32141900 | 0.41497400 |
| C | 1.54491700 | 7.46148400 | -1.49854300 |
| C | 1.74106600 | 6.37165300 | -2.36083400 |
| H | 1.21516400 | 6.31927300 | -3.31066000 |
| C | 2.63905100 | 5.35192100 | -2.03089700 |
| H | 2.79648800 | 4.54610500 | -2.74182200 |
| N | 5.07090900 | -1.16872200 | -1.68832200 |
| C | 7.38197800 | -0.29599700 | -1.40761100 |
| H | 7.46030300 | 0.65866600 | -1.94193400 |
| H | 8.37447600 | -0.75886200 | -1.45591800 |
| C | 6.35645500 | -1.16426400 | -2.13229500 |
| C | 6.72287200 | -1.88486900 | -3.26924500 |
| C | 5.75970400 | -2.60568600 | -3.96925500 |
| C | 4.42661800 | -2.63337800 | -3.51618500 |
| C | 4.14304200 | -1.89758600 | -2.35995500 |
| C | 3.38738800 | -3.42985900 | -4.25338400 |
| C | 3.18710900 | -3.23076400 | -5.63289700 |
| H | 3.74622200 | -2.46417800 | -6.16173200 |
| C | 2.27353200 | -4.00768600 | -6.34938400 |
| H | 2.16215700 | -3.80093300 | -7.41284600 |
| C | 1.54609700 | -5.02911600 | -5.71187800 |
| C | 1.74245600 | -5.23156500 | -4.33702900 |
| H | 1.21683000 | -6.02834200 | -3.81707800 |

| | | | | | | | |
|---|---|---|---|---|---|---|---|
| C | 2.64027700 | -4.43604400 | -3.61859800 | H | 0.25684600 | 8.41940700 | -2.94031100 |
| H | 2.79788700 | -4.64925500 | -2.56539300 | H | -0.25479500 | -4.40579500 | -7.75367300 |
| N | 1.48604600 | -6.73407000 | -7.52883900 | H | -1.87016400 | -6.93052900 | -7.61479900 |
| N | -1.48242400 | -6.08586800 | -8.06192500 | H | -1.02678600 | -6.37610000 | -8.94147900 |
| N | -1.48665900 | -3.94026700 | 9.30021900 | H | 0.25831300 | -6.75680500 | -5.82093300 |
| N | 1.48155700 | -3.15411600 | 9.59630000 | H | 1.87403700 | -6.13187900 | -8.27082900 |
| N | 1.48471900 | 9.88734300 | -2.06720900 | H | 1.03042400 | -7.54071400 | -7.98402100 |
| N | -1.48349000 | 10.02510900 | -1.23838200 | H | 2.30596600 | -7.13049000 | -7.03981600 |
| H | -3.13633400 | -1.13966900 | 2.45503700 | H | -2.30249600 | -5.52952600 | -8.35607200 |
| H | -3.13490400 | -1.55675500 | -2.21551100 | H | -2.30647500 | -4.47332000 | 8.96508000 |
| H | 7.75554300 | -1.88326100 | -3.60727300 | H | 2.30132200 | -2.53227300 | 9.69556400 |
| H | -3.13539100 | 2.69689200 | -0.24124200 | H | 2.30458600 | 9.66187600 | -2.65503800 |
| Zn | 4.96866500 | 0.00003000 | 0.00104600 | H | -2.30336800 | 10.00168700 | -0.60925000 |
| Zn | -4.96869000 | 0.00031800 | -0.00127500 | H | -6.05364100 | -1.43046200 | 5.61444500 |
| H | -0.25855700 | -4.51277000 | 7.69125800 | H | -7.75597500 | -0.61594800 | 4.02191100 |
| H | -1.87482600 | -3.13092900 | 9.80817800 | H | 6.05023800 | -2.61829400 | 5.17157500 |
| H | -1.03111100 | -4.55688900 | 9.99139200 | H | 3.13485700 | -0.75667900 | 2.60017400 |
| H | 0.25399900 | -1.66369400 | 8.76172000 | H | -6.05064600 | -4.14870300 | -4.04782700 |
| H | 1.86977000 | -4.09770100 | 9.44578400 | H | -7.75395100 | -3.17689900 | -2.54717900 |
| H | 1.02550000 | -3.14522500 | 10.52225400 | H | 3.13608600 | -1.87275300 | -1.95469700 |
| H | -1.87155400 | 10.06040100 | -2.19331000 | H | 6.05305500 | -3.16849400 | -4.85114600 |
| H | -1.02767800 | 10.93186800 | -1.04997700 | H | -6.05120700 | 5.57918200 | -1.57075400 |
| H | -0.25579700 | 8.91770700 | 0.06208800 | H | -7.75411100 | 3.79326400 | -1.48056100 |
| H | 1.87280600 | 10.22910800 | -1.17484500 | H | 3.13577800 | 2.62982500 | -0.64333900 |
| H | 1.02903400 | 10.68471000 | -2.53839500 | H | 6.05197600 | 5.78673400 | -0.31645700 |

## References


[S1] Begato, F.; Penasa, R.: Wurst, K.; Licini, G.; Zonta, C. Combining Imine Condensation Chemistry with [3,3] Diaza-Cope Rearrangement for One-Step Formation of Hydrolytically Stable Chiral Architectures. *Angew. Chem. Int. Ed.* **2023**, *62*, e202304490.

[S2] Kuzmic, P. Program DYNAFIT for the Analysis of Enzyme Kinetic Data: Application to HIV Proteinase. *Anal. Biochem*. **1996**, *237*, 260-273.

[S3] Gaussian 16, Revision C.01, Frisch, M. J.; Trucks, G. W.; Schlegel, H. B.; Scuseria, G. E.; Robb, M. A.; Cheeseman, J. R.; Scalmani, G.; Barone, V.; Petersson, G. A.; Nakatsuji, H.; Li, X.; Caricato, M.; Marenich, A. V.; Bloino, J.; Janesko, B. G.; Gomperts, R.; Mennucci, B.; Hratchian, H. P.; Ortiz, J. V.; Izmaylov, A. F.; Sonnenberg, J. L.; Williams-Young, D.; Ding, F.; Lipparini, F.; Egidi, F.; Goings, J.; Peng, B.; Petrone, A.; Henderson, T.; Ranasinghe, D.; Zakrzewski, V. G.; Gao, J.; Rega, N.; Zheng, G.; Liang, W.; Hada, M.; Ehara, M.; Toyota, K.; Fukuda, R.; Hasegawa, J.; Ishida, M.; Nakajima, T.; Honda, Y.; Kitao, O.; Nakai, H.; Vreven, T.; Throssell, K.; Montgomery, J. A., Jr.; Peralta, J. E.; Ogliaro, F.; Bearpark, M. J.; Heyd, J. J.; Brothers, E. N.; Kudin, K. N.; Staroverov, V. N.; Keith, T. A.; Kobayashi, R.; Normand, J.; Raghavachari, K.; Rendell, A. P.; Burant, J. C.; Iyengar, S. S.; Tomasi, J.; Cossi, M.; Millam, J. M.; Klene, M.; Adamo, C.; Cammi, R.; Ochterski, J. W.; Martin, R. L.; Morokuma, K.; Farkas, O.; Foresman, J. B.; Fox, D. J. Gaussian, Inc., Wallingford CT, **2016**.

[S4] Raulin, M.; Droghetti, F.; Zeppilli, D.; Begato, F.; Mondal, P. K.; Rancan, M.; Licini, G.; Orian, L.; Natali, M.; Zonta, C. Confined Metal Centers in a Symmetric Cage: Mono- and Heterodinuclear Complexes for Photocatalytic Hydrogen Evolution. *Inorg. Chem. Front.* **2026**, DOI: 10.1039/D5QI02424B.

[S5] Autschbach, J. Computing Chiroptical Properties with First-Principles Theoretical Methods: Background and Illustrative Examples. *Chirality* **2009**, *21*, E116-E152.
